# Antisense oligonucleotides targeting lncRNA AC104041.1 induces antitumor activity through Wnt2B/β-catenin pathway in head and neck squamous cell carcinomas

**DOI:** 10.1038/s41419-020-02820-3

**Published:** 2020-08-13

**Authors:** Mengwei Li, Xu Ding, Yinan Zhang, Xin Li, Haoze Zhou, Li Yang, Yilin Li, Peiwei Yang, Xiaomin Zhang, Jialiang Hu, Edouard Nice, Heming Wu, Hanmei Xu

**Affiliations:** 1 The Engineering Research Center of Peptide Drug Discovery and Development, China Pharmaceutical University, Nanjing, 210009 P.R. China; 2 State Key Laboratory of Natural Medicines, China Pharmaceutical University, Nanjing, 210009 P.R. China; 3 Department of Oral and Maxillofacial Surgery, Affiliated Hospital of Stomatology, Nanjing Medical University, Nanjing, 210029 P.R. China; 4 Department of Biochemistry and Molecular Biology, Monash University, Clayton, Melbourne, Victoria 3800 Australia

**Keywords:** Targeted therapies, Head and neck cancer

## Abstract

Long non-coding RNAs (lncRNAs) contribute to the initiation and progression of various tumors, including head and neck squamous carcinoma (HNSCC), which is a common malignancy with high morbidity and low survival rate. However, the mechanism of lncRNAs in HNSCC tumorigenesis remains largely unexplored. In this work, we identified a novel lncRNA AC104041.1 which is highly upregulated and correlated with poor survival in HNSCC patients. Moreover, AC104041.1 overexpression significantly promoted tumor growth and metastasis of HNSCC in vitro and in vivo. Mechanistically, AC104041.1 mainly located in the cytoplasm and could function as ceRNA (competing endogenous RNA) for miR-6817-3p, thereby stabilized Wnt2B, and consequently inducing β-catenin nuclear translocation and activation. Moreover, we demonstrate that salinomycin, which as a highly effective antibiotic in the elimination of cancer stem cells through the Wnt/β-catenin signaling, could enhance the inhibition of tumor growth by antisense oligonucleotides (ASO) targeting AC104041.1 in HNSCC cells and PDXs (patient-derived xenograft) model. Thus, our data provide preclinical evidence to support a novel strategy of ASOs targeting AC104041.1 in combination with salinomycin and may as a beneficial treatment approach for HNSCC.

## Introduction

Head and neck squamous cell carcinoma (HNSCC) is the seventh most common cancer, with a yearly incidence of 890,000 and a death rate of 450,000 patients in the world^1^. Most HNSCC patients are diagnosed at an advanced stage of the disease when prognosis is poor, and the 5-year survival rate is only 50%^2,3^. Therefore, the discovery of novel biomarkers to facilitate the early detection of HNSCC and bolster the chances for positive patient outcomes is a high priority.

Long non-coding RNAs (lncRNAs) are transcripts longer than 200 nucleotides and closely linked to cancer initiation and progression via diverse mechanisms^4^. Some lncRNAs act as epigenetic regulators which involve chromatin modulation^5^, DNA organization^6^, and Histone modification^7^, and other lncRNAs (such as LINK-A, EPIC1 and LDLRAD4-AS1) could interact with RNA or proteins to modulate gene expression^8–10^. However, it largely remains unknown with regards to a variety of novel lncRNAs in the pathological regulation of HNSCC.

Antisense oligonucleotides (ASOs) are synthetic single-stranded DNA analogs with 16–22 bases which bind to target RNAs (including mRNAs and non-coding RNAs) and led to endonuclease mediated transcript knockdown^11^. Recently, ASOs have been developed as pharmacological agents to treat cancer, such as Floxuridin-integrated ASO with anti-Bcl-2 to reverse chemoresistance in hepatocellular carcinoma^12^, and anti-multiple myeloma activity of LNA gapmeR ASO targeting lncRNA MALAT1^13^. The current promising results of the ASO-based therapy promote us to explore new strategies in the treatment of HNSCC.

In this study, we identify an oncogenic lncRNA, AC104041.1 which is highly expressed in HNSCC tissues and critical for HNSCC growth and metastasis. Lnc-AC104041.1 attenuates miR-6817-3p targeting of the Wnt family ligand Wnt2B, thus maintaining Wnt2B stability and strengthening Wnt/β-catenin signaling. Here we report the first evidence of the anti-tumor activity of ASO-targeting AC104041.1 in vitro and in vivo, which is enhanced by salinomycin (Sal) that inhibits Wnt signaling^14^. Taken together, our finding reveal the important role of lnc-AC104041.1/Wnt2B/ β-catenin axis in tumorigenesis of HNSCC, which can be used as predictive biomarkers as well as potential targets for developing significant therapeutic advances in HNSCC.

## Results

### LncRNA AC104041.1 is highly expressed and correlated with poor survival in HNSCC patients

**Fig. 1:**
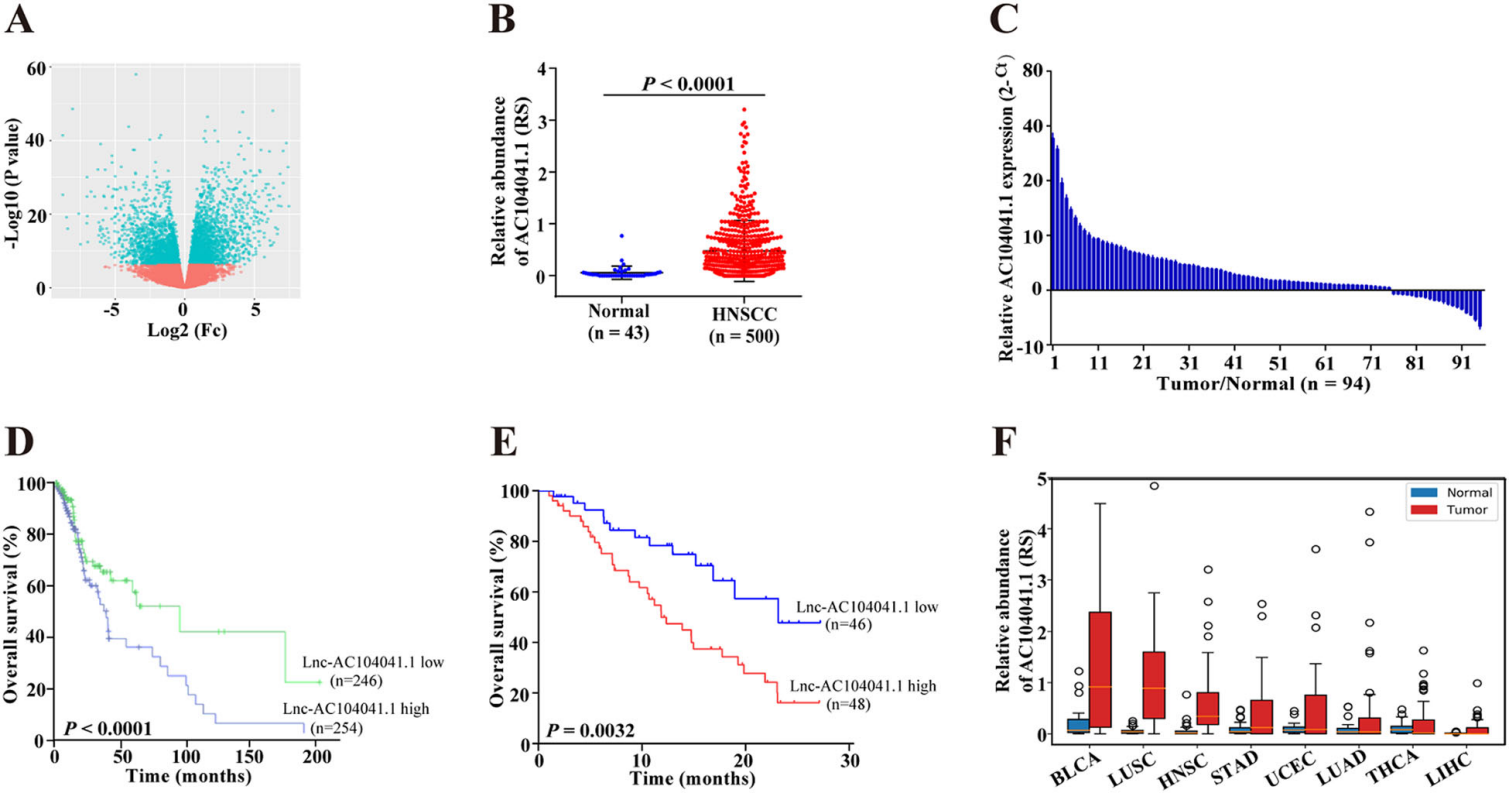
Identification of lnc-AC104041.1 and its relationship to HNSCC patients. **a** Volcano plot presenting the differential lncRNAs of HNSCC patients from the TCGA dataset. **b** Differential expression of AC104041.1 in HNSCC (*n* = 500) and normal tissues (*n* = 43) from the TCGA database. Each symbol represents one patient, ***P < 0.001 (one-way ANOVA). **c** Expression of AC104041.1 in freshly removed HNSCC tumor tissues and paired adjacent normal tissues (*n* = 94). Each symbol shows the average of triplicate qPCR results from one patient. Kaplan–Meier analysis of AC104041.1 expression levels with overall survival (OS) of HNSCC patients in TCGA database (**d**) and independent cohorts (**e**). Higher expression of AC104041.1 is significantly correlated with poor OS (*P* < 0.01, log-rank test). **f** AC104041.1 expression of different cancer types in the TCGA database. BLCA bladder urothelial carcinoma, LUSC lung squamous cell carcinoma, HNSC head and neck squamous cell carcinoma, STAD stomach adenocarcinoma, UCEC uterine corpus endometrial carcinoma, LUAD lung adenocarcinoma, THCA thyroid carcinoma, LIHC liver hepatocellular carcinoma.

To interrogate deregulated lncRNAs in HNSCC, we developed an integrated computational pipeline to analyse 500 HNSCC samples from TCGA datasets which include both lncRNAs expression (Fig. S1A). A final set of 563 differentially expressed lncRNAs in HNSCC compared with normal tissues (|fold change| >2, *P* value < 0.05 and false-discovery rate (FDR) <0.01) were identified and clustered based on Pearson correlation (Fig. 1a). Because patients survival represents a key clinical index of tumor aggressiveness^15^, to explore the clinical relevance of the 563 differentially lncRNAs identified above, we investigated the correlations of top 30 upregulation lncRNAs (Table S1) with overall survival (OS) in HNSCC patients. The 500 samples were randomly and equally divided into a training set (*n* = 250) and a validation set (*n* = 250), we performed univariate Cox Kaplan–Meier analysis and found that 12 lncRNAs were significantly related to overall survival in the training set. Furthermore, eight of these 12 lncRNAs were significantly correlated with poor overall survival in the validation set (Table S2).

To assess the predictive accuracy of these deregulated lncRNAs, ROC analysis was performed to evaluate the sensitivity and specificity of survival prediction. The AUC was calculated using 5 years as the cut-off survival time, four lncRNAs (AC104041.1, LINC00460, LINC00958, and ST3GAL4-AS1) showed AUC values above 0.6, with AC104041.1 producing the highest AUC value (Table S3, Fig. S1B). Consistent with the analysis results of TCGA database (Fig. 1b), AC104041.1 expression was significantly higher in 94 clinical fresh HNSCC samples (which as the independent cohort and the clinical information listed in Table S4) than that in adjacent normal tissues (Fig. 1c). Moreover, high AC104041.1 expression was associated with decreased patient overall survival in both TCGA and independent cohorts (Fig. 1d, e), univariate and multivariate Cox regression analysis revealed that AC104041.1 expression, clinicopathological stage and TNM status were independent prognostic factors in HNSCC patients from TCGA cohort (Table S5). In addition, bioinformatics analysis using CPAT probability, phastCons score^16^ and ORF finder indicated that AC104041.1 had no protein-coding capacity (Fig. S1C, D). Strikingly, AC104041.1 was highly expressed in different kinds of solid tumors from TCGA database (Fig. 1f), which indicated that AC104041.1 generally acts as an oncogene.

### AC104041.1 functions as a potential oncogenic lncRNA by promoting HNSCC growth and metastasis

**Fig. 2:**
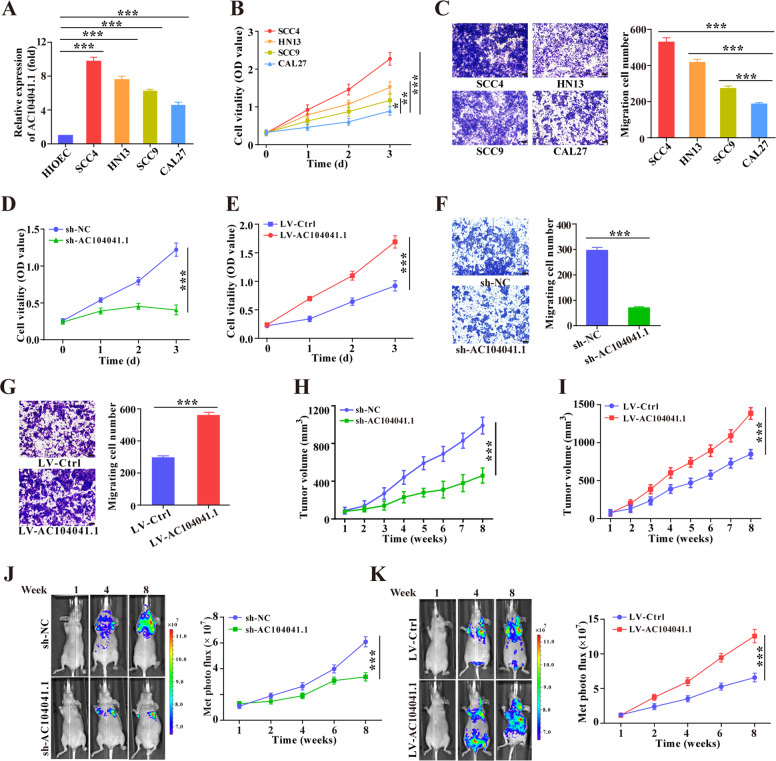
lnc-AC104041.1 acts as an oncogenic driver for HNSCC cells. **a** qRT-PCR analysis of AC104041.1 in HNSCC cells and normal epithelial cell. Data are mean values ± SD, the experiment was performed in triplicates and repeated three times, ***P < 0.001 (Student’s *t*-test). **b** The proliferation ability was compared in four different HNSCC cells (*n* = 5). Data are mean values ± SEM, the experiment was repeated three times, *P < 0.05, **P < 0.01, ***P < 0.001 (two-way ANOVA). **c** The migration ability was compared in four different HNSCC cells. Data are mean values ± SD, the experiment was performed in triplicates and repeated three times, ***P < 0.001 (Student’s *t*-test). MTT proliferation assay of SCC4 cells with AC104041.1 knockdown (**d**) or CAL27 cells with AC104041.1 overexpression (**e**). *n* = 5, the experiment was repeated three times. Data are presented as the mean values ± SEM, ***P < 0.001, compared with control cells (two-way ANOVA). Migration assay in SCC4 cells with knockdown (**f**) or CAL27 cells with overexpression (**g**) of AC104041.1. Data are presented as the mean values ± SD, the experiment was performed in triplicates and repeated three times, ***P < 0.001 (Student’s *t*-test). Tumor growth in Balb/c nude mice 8 weeks after subcutaneous inoculation of SCC4 cells with AC104041.1 knockdown (**h**) or CAL27 cells with AC104041.1 overexpression (**i**), *n* = 5 mice for each group. Data are presented as the mean values ± SEM, ***P < 0.001 (two-way ANOVA). Bioluminescence signal of SCC4 cells with AC104041.1 knockdown (**j**) or CAL27 cells with AC104041.1 overexpression (**k**) detected using an in vivo imaging system for 8 weeks (*n* = 5 mice for each group). Data are presented as the mean values ± SD, ***P < 0.001 (two-way ANOVA).

To ascertain the function of AC104041.1 in HNSCC, we measured AC104041.1 expression in normal oral epithelial cell line (HIOEC) and four HNSCC cell lines. As shown in Fig. 2a, compared with normal HIOEC cells, SCC4 cells harbored the highest AC104041.1 levels, while CAL27 cells had the lowest AC104041.1 levels (Fig. 2a). Consistently, SCC4 cells showed higher cell proliferation and migration ability than CAL27 cells (Fig. 2b, c). Then, we stably silenced AC104041.1 via shRNA in SCC4 cells (Fig. S2A), which led to significantly decreased cell proliferation, migration, and colony formation (Fig. 2d, f and Fig. S2C), whereas AC104041.1 overexpression (Fig. S2B) significantly increased the HNSCC cell malignant abilities (Fig. 2e, g and Fig. S2D). Furthermore, to assess the effects of AC104041.1 on the tumor growth and metastasis of HNSCC in vivo, we generated two different xenograft models with AC104041.1 stable knockdown or overexpression cells. Eight weeks post-engraftment, AC104041.1-depleted SCC4 xenografts showed a significant decrease in the growth parameters compared with the control xenografts (Fig. 2h), contrasting findings were observed in the AC104041.1 overexpression cell xenograft model (Fig. 2i), indicating that AC104041.1 is positively correlated with tumorigenesis (Fig. S2E). Moreover, the impact of AC104041.1 on HNSCC metastasis was evaluated by AC104041.1 knockdown SCC4 cells or overexpression CAL27 cell with injection into the tail vein of nude mice. Knockdown of AC104041.1 significantly inhibited lung metastasis (Fig. 2j and Fig. S2F, G), while overexpression of AC104041.1 induced more metastatic foci for a period of eight weeks (Fig. 2K and Fig. S2F, H). Taken together, our results uncover the oncogenic activity of AC104041.1 in modulating tumor growth and metastasis.

### AC104041.1 functions as a competing endogenous RNA by directly binding to miR-6817-3p

**Fig. 3:**
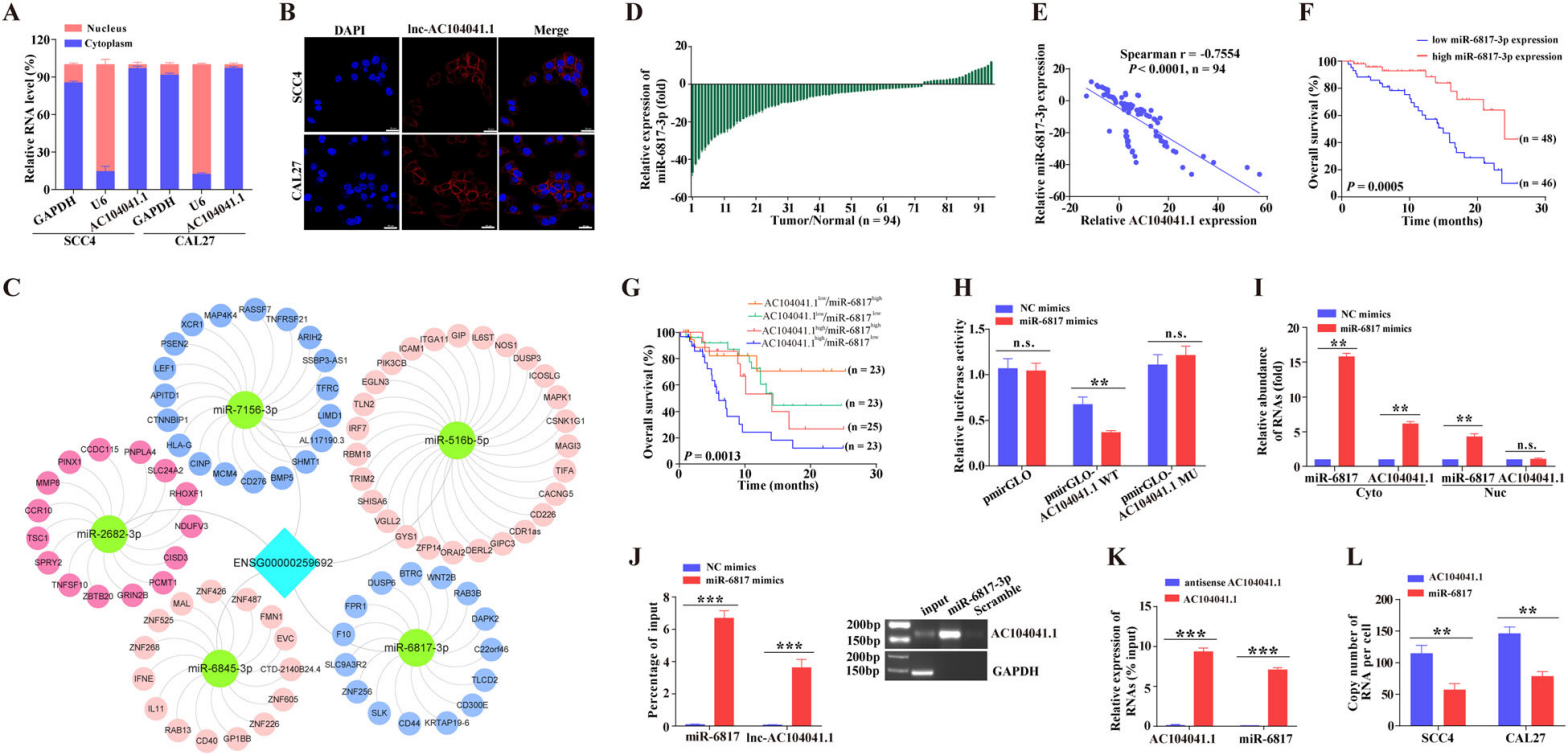
Lnc-AC104041.1 interacts with miR-6817-3p to regulate HNSCC cell proliferation and migration. **a** Relative AC104041.1 expression levels in the cytoplasmic (cyto) and nuclear (nuc) fractions of SCC4 and CAL27 cells. U6 and GAPDH expression levels were measured as positive controls. Data are % distribution calculated to complete amount of transcript, the experiment was performed in triplicates and repeated three times. **b** The subcellular distribution of AC104041.1 was visualized by FISH assay in SCC4 and CAL27 cells. **c** A scheme of miRNA target network showing the relationship of AC104041.1 downregulated miRNAs and their predicted target mRNAs. **d** Relative expression levels of miR-6817-3p in HNSCC tissues compared with their matched non-tumorous adjacent tissues (*n* = 94). Each symbol shows the average of triplicate qPCR results from one patient. **e** Negative correlation between AC104041.1 and miR-6817-3p levels were measured in the same set of patients by Spearman correlation analysis (*n* = 94, Spearman correlation *r* = −0.7554, *P* < 0.001). Each dot represents the average of qPCR results performed in triplicates. **f** Kaplan–Meier analysis of the correlation between miR-6817-3p expression levels and overall survival. Lower expression of miR-6817-3p is significantly correlated with poor OS (***P < 0.001, log-rank test). **g** Association of AC104041.1 and miR-6817-3p with overall survival in HNSCC patients (**P < 0.01, log-rank test). **h** Luciferase activities were measured in SCC4 cells co-transfected with luciferase reporters containing nothing, AC104041.1 or mutant transcript and miR-6817-3p mimics. Data are presented as the mean values ± SEM, the experiment was performed in triplicates and repeated three times, ***P < 0.001, compared with control cells (one-way ANOVA). **i** Biotin-labeled miR-6817-3p pulled down AC104041.1 in the cytoplasmic but not the nuclear fraction of SCC4 cells. Data are presented as the mean values ± SEM, the experiment was performed in triplicates and repeated three times, **P < 0.01, compared with control cells (one-way ANOVA). **j** AC104041.1 was pulled down after transfecting with biotin-miR-6817-3p in SCC4 cells. Data are presented as the mean values ± SD, the experiment was performed in triplicates and repeated three times, ***P < 0.001 (Student’s *t*-test). **k** miR-6817-3p was pulled down with biotin-AC104041.1 probe in SCC4 cells. Data are mean values ± SEM, the experiment was performed in triplicates and repeated three times, ***P < 0.001, compared with control cells (one-way ANOVA). **l** Quantitation of copy numbers of AC104041.1 and miR-6817-3p in HNSCC cells. Data are presented as the mean values ± SD, the experiment was performed in triplicates and repeated three times, **P < 0.01 (Student’s *t*-test).

The functionality of lncRNAs in part begins with cellular localization: nuclear lncRNAs are involved in chromatin interaction, transcriptional regulation, and RNA processing, and cytoplasmic lncRNAs can modulate mRNA stability or translation and influence cellular signaling cascades^17^_._ The qRT-PCR of cytosolic and nuclear fractions from HNSCC cells indicated that AC104041.1 is mainly located in the cytoplasm (Fig. 3a), which was confirmed by RNA fluorescence in situ hybridization of AC104041.1 (Fig. 3b). Thus, we hypothesized that AC104041.1 could function as a ceRNA, acting as a molecular sponge for miRNAs^18^. Subsequently, we devised an integrated computational approach that investigated whether AC104041.1 serves as sp-lncRNA (Fig. S3A), the results showed that sponge regulatory network of AC104041.1 contained 5 miRNAs (miR-7156-3p, miR-6845-3p, miR-6817-3p, miR-516b-5p, and miR-2682-3p) and 809 mRNAs in total that were significantly associated (Fig. 3c). Furthermore, the functional enrichment analysis showed that target mRNAs of these miRNAs were significantly enriched in pathways related to cancer, including cell cycle, cell apoptosis, cell adhesion and actin cytoskeleton regulation (Fig. S3B), which are consistent with our previous observation that the essential role of AC104041.1 might be functionally engaged with vital cellular processes. To identify the correlation of lnc-AC104041.1 with the five miRNAs, the expression of these miRNAs was assessed in 25 pairs of HNSCC/adjacent normal tissues. Three (miR-7156-3p, miR-6817-3p, and miR-516b-5p) of these five miRNAs were significantly downregulated in HNSCC tissues (Fig. S3C), and miR-6817-3p exhibited the highest negative correlation with AC104041.1 (Table S6), further analysis showed that miR-6817-3p expression was markedly decreased (Fig. 3D) and negatively correlated with AC104041.1 in 94 HNSCC tissues (Fig. 3e). Moreover, kaplan–Meier analysis revealed that high miR-6817-3p expression was significantly correlated with favorable overall survival (Fig. 3f). In contrast, patients whose tumors exhibit downregulation of miR-6817-3p and increased AC104041.1 expression have the poorest overall survival (Fig. 3g), indicating that combined AC104041.1 and miR-6817-3p investigation might improve the accuracy of clinical prognosis for HNSCC.

A search of the TargetScan (http://www.targetscan.org/) prediction algorithm^19^ showed that AC104041.1 contained highly complementary regions (seven bases) to the ‘seed’ region of miR-6817-3p. To confirm the interaction of AC104041.1 and miR-6817-3p, we subcloned full length AC104041.1 (AC104041.1-WT) or the mutated complementary miR-6817-3p-BRs (AC104041.1-MU) into the pmirGLO dual-luciferase reporter vector (Fig. S3D). Notably, introduction of miR-6817-3p mimics or inhibitors (Fig. S3E) selectively reduced or alternatively increased luciferase activity of AC104041.1-WT, respectively, whereas the mutation of the seed region (AC104041.1-MU) abolished the miR-6817-3p-dependent suppression of luciferase activity (Fig. 3h and Fig. S3F). Seeking further evidence for this direct interaction, we showed that in vitro-synthesized miR-6817-3p mimics precipitated endogenous AC104041.1 from purified cytoplasmic but not nuclear fractions (Fig. 3i), indicating that the AC104041.1 and miR-6817-3p interaction occurs largely in the cytoplasm. Meanwhile, RNA pull-down assay with biotin-miR-6817-3p or biotin-AC104041.1 sequence in SCC4 cells suggested that endogenous AC104041.1 had direct association with miR-6817-3p (Fig. 3j, k). Furthermore, to function as a ceRNA, the relative abundance of AC104041.1 and miR-6817-3p should be comparable^20^, the absolute copy number of AC104041.1 and miR-6817-3p per cell indicated that the copies per cell in AC104041.1 were significantly higher than those of miR-6817-3p in both SCC4 and CAL27 cells (Fig. 3l). All these data collectively suggest that AC104041.1 plays a pivotal role in promoting HNSCC cell proliferation and migration through directly binding to miR-6817-3p.

### AC104041.1 functions as a ceRNA for miR-6817-3p to facilitate Wnt2B expression

**Fig. 4:**
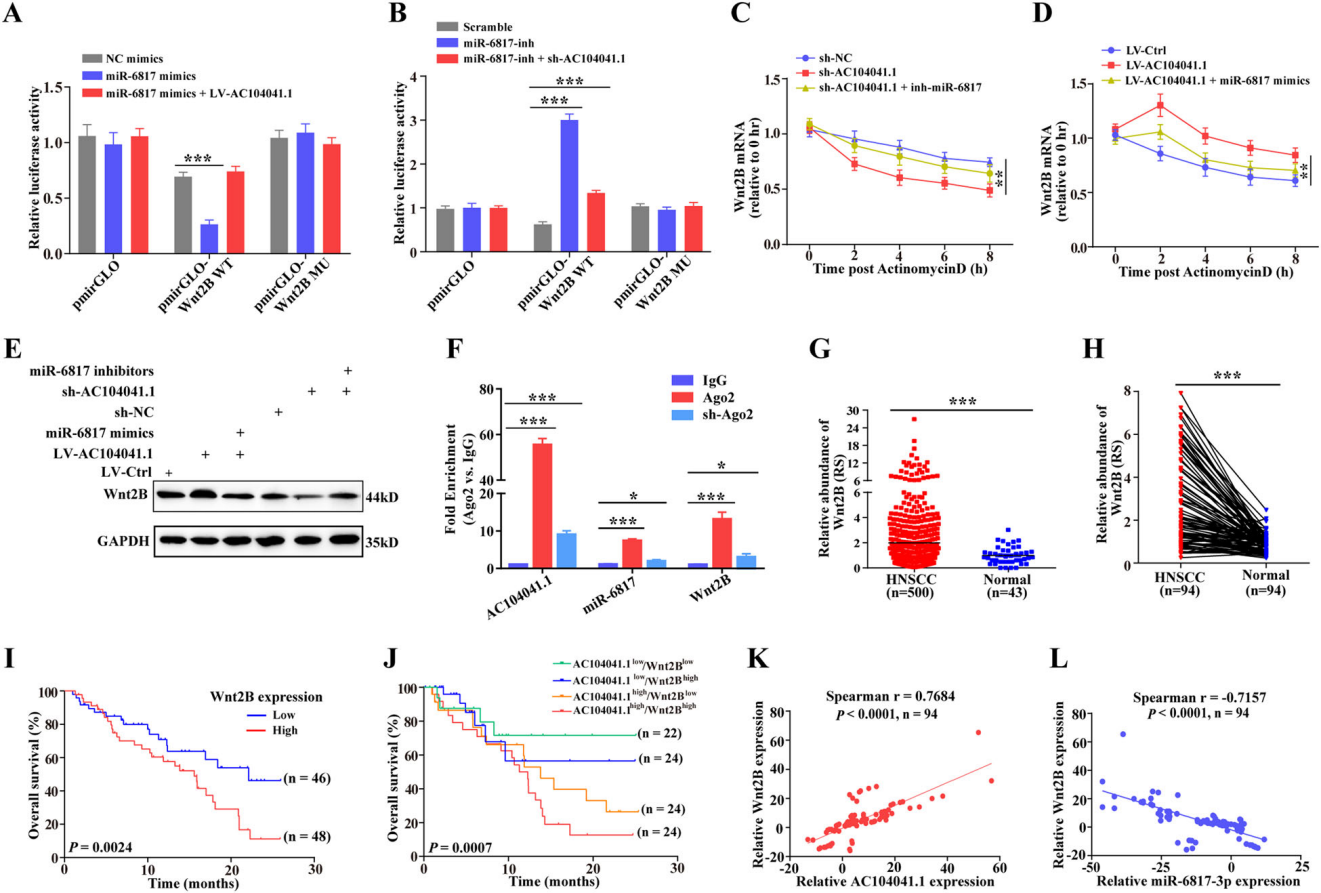
AC104041.1 competes with miR-6817-3p to sustain Wnt2B expression in HNSCC. **a** Luciferase activity in SCC4 cells co-transfected with luciferase reporter containing Wnt2B and miR-6817-3p mimics or in combination with AC104041.1 overexpression. Data are presented as the mean values ± SEM, the experiment was performed in triplicates and repeated three times, ***P < 0.001, compared with control cells (one-way ANOVA). **b** Luciferase activity in SCC4 cells co-transfected with luciferase reporter containing Wnt2B and miR-6817-3p inhibitors or in combination with AC104041.1 knockdown. Data are presented as the mean values ± SEM, the experiment was performed in triplicates and repeated three times, ***P < 0.001, compared with control cells (one-way ANOVA). **c** Downregulation of Wnt2B mRNA stability caused by silencing of AC104041.1 was reversed by miR-6817-3p inhibitors. Data are mean values ± SEM, the experiment was performed in triplicates and repeated three times, **P < 0.01 (two-way ANOVA). **d** Upregulation of Wnt2B mRNA stability caused by overexpression of AC104041.1 was reversed by miR-6817-3p mimics. Data are mean values ± SEM, the experiment was performed in triplicates and repeated three times, **P < 0.01 (two-way ANOVA). **e** Immunoblotting for Wnt2B levels in empty-vector and ectopic AC104041.1-expressing SCC4 cells in the presence of miR-6817-3p mimics or knockdown of AC104041.1 cells in the presence of miR-6817-3p inhibitors. **f** AGO2 competing assay using AGO2 antibody-mediated RNA-IP in the indicated groups. RNA levels of precipitated AC104041.1, miR-6817-3p, and Wnt2B are tested using qPCR assay. Data are presented as the mean values ± SEM, *P < 0.05, ***P < 0.001 (one-way ANOVA). **g** Relative expression of Wnt2B in HNSCC tissues compared with their matched non-tumorous adjacent tissues in TCGA cohorts (*n* = 500). Each symbol represents one patient. ***P < 0.001 (Student’s *t*-test). **h** Relative expression levels of Wnt2B in paired normal and HNSCC tissues (*n* = 94). Each dot represents the average of qPCR results performed in triplicates. Horizontal lines represent mean values. ***P < 0.001 (paired *t*-test). **i** Kaplan–Meier analysis of the correlation between Wnt2B expression levels and overall survival. Higher expression of Wnt2B is significantly correlated with poor OS (**P < 0.01, log-rank test). **j** Association of AC104041.1 and Wnt2B with overall survival in HNSCC. **k** Positive correlation between AC104041.1 and Wnt2B levels in HNSCC tissues (*n* = 94, Spearman correlation *r* = 0.7684, *P* < 0.001). Each dot represents the average of qPCR results performed in triplicates. **l** Negative correlation between miR-6817-3p and Wnt2B levels in HNSCC tissues (*n* = 94, Spearman correlation *r* = −0.7157, *P* < 0.001). Each dot represents the average of qPCR results performed in triplicates.

Among the sponge network of AC104041.1, there are many predicted targets of miR-6817-3p (Table S7), luciferase reporter assays with different wide-type seed region for each of the top ten candidate targets were performed to explore the bona fide targets (Fig. S4A-C), the luciferase activity of pmirGLO-Wnt2B WT reporter was decreased mostly with transfected miR-6817-3p mimics, and ectopic expression of AC104041.1 rescued the luciferase activity inhibition of miR-6817-3p (Fig. 4a and Fig. S4D). Reciprocally, the depletion of miR-6817-3p increased the luciferase activity of pmirGLO-Wnt2B WT, which was abolished by inhibition of AC104041.1 (Fig. 4b), so we postulated Wnt2B as the direct target of miR-6817-3p. Furthermore, we blocked new RNA synthesis with actinomycin D and strikingly found that co-introduction of anti-miR-6817-3p abolished the inhibitory effect of AC104041.1 knockdown on Wnt2B mRNA and protein expression (Fig. 4c, e), whereas co-introduction of miR-6817-3p mimics decreased the upregulation of AC104041.1 overexpression on Wnt2B mRNA stability and protein levels (Fig. 4d, e), indicating that AC104041.1 functions to regulate Wnt2B mRNA stability by sequestering miR-6817-3p.

A critical regulatory mechanism of cytoplasmic lncRNAs is their action as sponges to inhibit miRNA expression, thereby augmenting miRNA downstream gene expression in an RNA-induced silencing complex (RISC)-dependent manner (Du). Argonaute2 (Ago2) is a key component of the RISC that can directly degrade mRNA by a slicing mechanism^21,22^, we performed RIP assays using the AGO2 antibody to further confirm the interaction between AC104041.1, miR-6817-3p, and Wnt2B. The levels of AC104041.1, miR-6817-3p, and Wnt2B were significantly higher in AGO2 pull down than those in the IgG group (Fig. 4f). In addition, the relative enrichment (AGO2 IP/IgG IP) of AC104041.1, miR-6817-3p, and Wnt2B was significantly decreased with the knockdown of Ago2 (Fig. 4f and Fig. S4E), and the result of RNA pull-down assay with a biotin-AC104041.1 probe showed that Ago2 was pulled down by biotin-labeled AC104041.1 but not antisense RNA (Fig. S4F). Moreover, knockdown of AC104041.1 significantly decreased AC104041.1 levels and increased Wnt2B levels in AGO2 pull-down complex (Fig. S4G), indicating that miR-6817-3p could competitively bind AC104041.1 and Wnt2B in the Ago2-based miRNA-induced silencing complex (RISC).

Wnt2B was reported to play critical roles in different tumors cell proliferation, migration, and EMT process^23,24^, here we first indicated that Wnt2B was a direct target of miR-6817-3p. Interestingly, analysis of TCGA data showed that Wnt2B expression was broadly increased in HNSCC tissues (Fig. 4g). Indeed, our detailed clinical investigations demonstrated significantly elevated levels of Wnt2B (Fig. 4h) and its potential to independently predict clinical outcomes in HNSCC patients (Fig. 4i and Fig. S4H). Moreover, patients who exhibited upregulation of AC104041.1 and increased Wnt2B expression had the worst survival (Fig. 4j and Fig. S4I), Wnt2B expression was shown to be positively correlated with AC104041.1 (Fig. 4k and Fig. S4J) and negatively correlated with miR-6817-3p (Fig. 4l), suggesting that AC104041.1 stabilized Wnt2B mRNA by competitively binding to miR-6817-3p and its co-expression strongly affected HNSCC patient survival.

### AC104041.1 promotes HNSCC tumorigenesis through activation of Wnt2B/β-catenin pathway

**Fig. 5:**
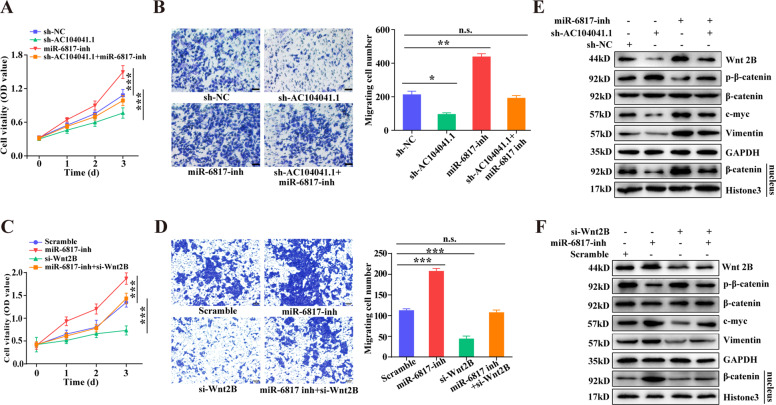
AC104041.1 promotes HNSCC tumorigenesis through activation of Wnt2B/β-catenin pathway. **a** Cell proliferation were assessed in AC104041.1 knockdown or combined with miR-6817-3p inhibitors (*n* = 5). Data are presented as the mean values ± SEM, the experiment was repeated three times, ***P < 0.001, compared with control cells (two-way ANOVA). **b** Cell migration assay were assessed in AC104041.1 knockdown or combined with miR-6817-3p inhibitors. Data are presented as the mean values ± SD, the experiment was performed in triplicates and repeated three times, *P < 0.05, **P < 0.01 (Student’s *t*-test). **c** Cell proliferation were assessed in miR-6817-3p inhibitors or combined with Wnt2B knockdown (*n* = 5). Data are presented as the mean values ± SEM, the experiment was repeated three times, ***P < 0.001, compared with control cells (two-way ANOVA). **d** Cell migration assay were assessed in miR-6817-3p inhibitors or combined with Wnt2B knockdown. Data are presented as the mean values ± SD, the experiment was performed in triplicates and repeated three times, *P < 0.05, **P < 0.01 (Student’s *t*-test). **e** Immunoblots of indicated proteins in SCC4 cells transfected with AC104041.1 knockdown or combined with miR-6817-3p inhibitors. **f** Immunoblots of indicated proteins in SCC4 cells transfected with miR-6817-3p inhibitors or combined with Wnt2B knockdown.

Based on the previous report that Wnt2B expression was involved in the Wnt/β-catenin signaling pathway, which has a key role in cancer metastasis, the EMT process and self-renewal of cancer stem cells^25^. We gain an insight into the correlation between AC104041.1/miR-6817-3p/Wnt2B axis and tumor growth and metastasis of HNSCC, introduction of AC104041.1 knockdown or miR-6817-3p inhibitors selectively reduced or increased HNSCC cell proliferation and migration, and inh-miR-6817-3p attenuated the inhibition of cell proliferation and migration mediated by AC104041.1 knockdown (Fig. 5a, b). Moreover, co-introduction of Wnt2B knockdown abolished the induced effect of inh-miR-6817-3p on HNSCC cell proliferation and migration (Fig. 5c, d). These results further indicate the important function of AC104041.1 sponge network in HNSCC tumorigenesis.

To our knowledge, the key switch in the canonical Wnt pathway is the protein β-catenin, upon Wnt pathway activation in the presence of Wnt ligands, the phosphorylated LRP receptor might act to directly inhibit GSK-3β and thereby promote β-catenin stabilization and nuclear accumulation^26^. Therefore, β-catenin forms an active complex with LEF (lymphoid enhancer factor) and TCF (T-cell factor) proteins^27^ and activates transcription of downstream target genes including c-myc, cyclin D1, vimentin, c-jun^28^, which are the basis for tumorigenesis. Strikingly, AC104041.1 knockdown resulted in marked reduction in Wnt2B, nuclear translocation of β-catenin, c-myc, and vimentin expression, which were diminished by miR-6817-3p inhibitors (Fig. 5e). As expected, Wnt2B knockdown also alleviated the increased effects of inh-miR-6817-3p on these above proteins expression (Fig. 5f). Collectively, these results suggested that AC104041.1 sponged miR-6817-3p, thereby releasing Wnt2B from miR-6817-3p and promoting Wnt/β-catenin pathway activation.

### Salinomycin promotes the anti-tumor activity of AC104041.1 specific LNA-ASO in vitro and in vivo

**Fig. 6:**
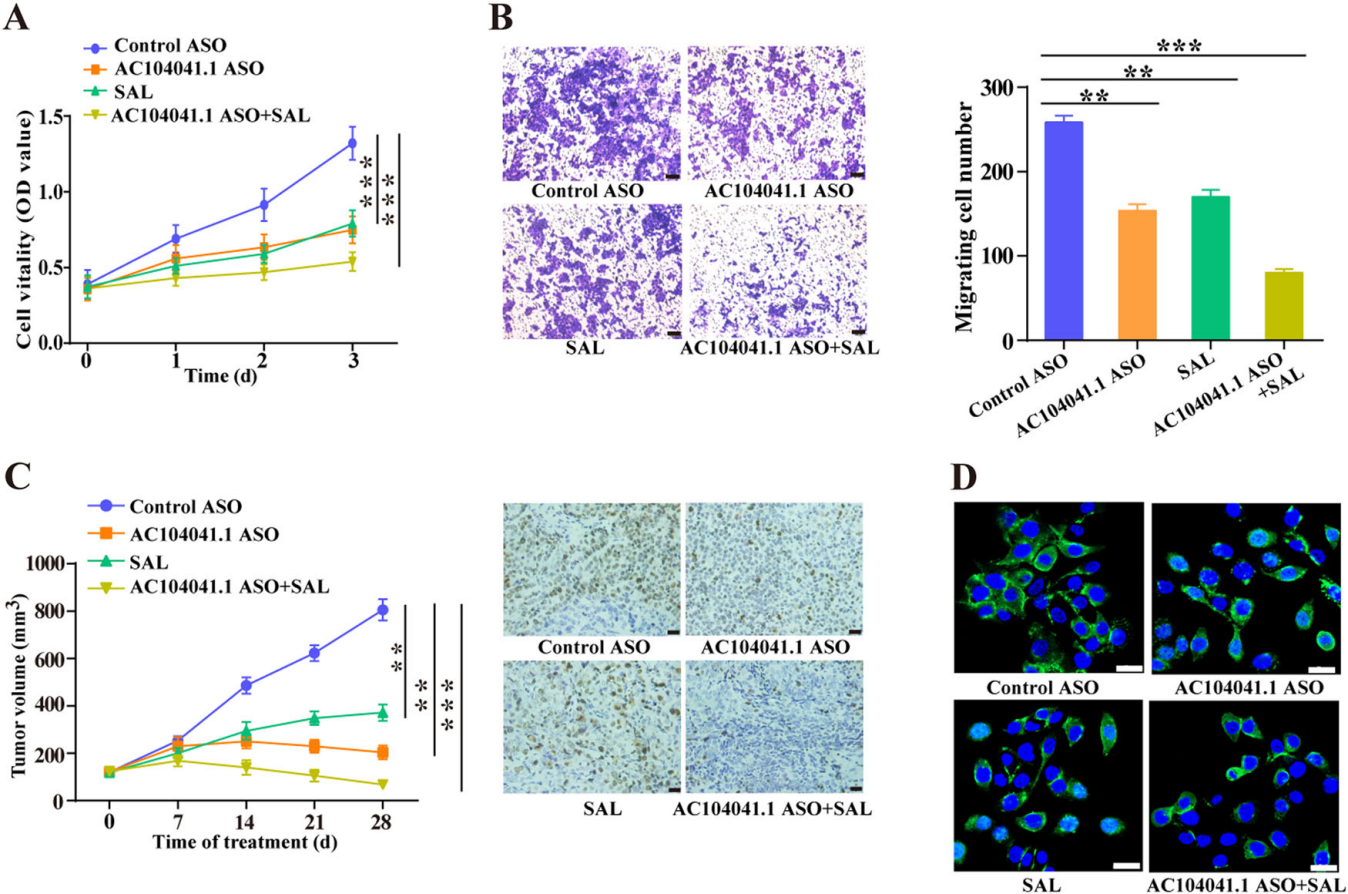
Salinomycin promotes the anti-tumor activity of AC104041.1 specific LNA-ASO in vitro and in vivo. **a** Cell proliferation assay of SCC4 cells transfected with AC104041.1 specific LNA-ASO or combined with salinomycin treatment (*n* = 5). Data are presented as the mean values ± SEM, the experiment was repeated three times, ***P < 0.001, compared with control cells (two-way ANOVA). **b** Cell migration assay of SCC4 cells transfected with AC104041.1 specific LNA-ASO or combined with salinomycin treatment. Data are presented as the mean values ± SD, the experiment was performed in triplicates and repeated three times, **P < 0.01, ***P < 0.001 (Student’s *t*-test). **c** Tumor growth in Balb/c nude mice and representative immunohistochemical images of Ki-67 from tumors after subcutaneous injection of AC104041.1 specific LNA-ASO or combined with salinomycin treatment (*n* = 5 mice for each group). Data are presented as the mean values ± SEM, **P < 0.01, ***P < 0.001 (two-way ANOVA). **d** Immunofluorescence of β-catenin (green) and nuclei (blue) in SCC4 cells transfected with AC104041.1 specific LNA-ASO or combined with salinomycin treatment. Scale bar represents 20 µm.

Since the first antisense oligonucleotide agent was approved by the US Food and Drug Administration (FDA) to treat cytomegalovirus (CMV) induced chorioretinitis^29^, which sparking high hopes for the potential therapy of this new class of drugs for human disease including cancer. To explore the promising druggable of AC104041.1, we designed locked nucleic acid (LNA)-modified antisense oligonucleotides (ASOs) with specificity for AC104041.1 (Fig. S5A–C) and evaluated its anti-tumor activity in vitro and in vivo. Meanwhile, salinomycin has been reported to kill cancer stem cell (CSC) as an inhibitor of Wnt signaling^30^. Therefore, we explored the combined inhibition of AC104041.1 specific LNA-ASO and salinomycin in HNSCC, either AC104041.1 LNA-ASO or salinomycin resulted in a significant inhibition of cell viability and migration in vitro (Fig. 6a, b) and tumor growth of patient-derived xenograft (PDX) models generated from HNSCC patients in vivo (Fig. 6c). Notably, upon salinomycin treatment, cell viability and migration showed a more pronounced response to AC104041.1 LNA-ASO (Fig. 6a, b), and tumor volume exhibited much smaller than either single agent (Fig. 6c). In addition, immunofluorescence staining showed that nuclear β-catenin signals was significantly lowest in cells treated with AC104041.1 LNA-ASO and salinomycin (Fig. 6d). Together, these data clearly indicated that AC104041.1 LNA-ASO could as a potential drug to suppress HNSCC tumorigenesis and combination of salinomycin effectively promoted its anti-tumor activity via inhibition of Wnt/β-catenin pathway.

## Discussion

Despite the vast majority of key lncRNAs in cancers, a few functional and mechanistic lncRNAs have been characterized. One important molecular mechanism of lncRNAs is their involvement in competing endogenous RNA (ceRNA) or “RNA sponges” with miRNAs to reduce the regulatory effect on target mRNA^31^. For instance, lncRNA PTAR as a ceRNA for the miR-101-3p regulates ZEB1 expression to promotes EMT and invasion-metastasis in ovarian cancer^32^, lncRNA- KRTAP5-AS1 and lncRNA-TUBB2A act as ceRNAs for miR-34 and miR-449 to facilitate CLDN4 expression and consequently promote gastric cancer cell proliferation and EMT process^33^. In this study, we first identified that lncRNA AC104041.1 is highly expressed in HNSCC and is associated with patient outcomes. By further integrating the gene expression profiling data of AC104041.1, miRNAs and mRNAs in tumor and the sequence features of RNAs, we showed that AC104041.1 functions as a ceRNA for miR-6817-3p in the cytoplasm to increase Wnt2B stability and activate the Wnt/β-catenin pathway. To the best of our knowledge, this is the first investigation of Wnt2B in a lncRNA mediated sponge regulatory network to elucidate its regulatory mechanisms in HNSCC cell proliferation and metastasis.

Antisense oligonucleotides (ASOs) were first discovered to specifically bind target RNA sequence and regulate protein expression, but how to translate these agents into the clinic is hampered by inadequate target engagement, insufficient biological activity, and off-target toxic effects^34^. In recent years, novel chemical modifications of ASOs have been employed to address these issues. For example, Ribose substitutions, including 2′-O-methoxyethyl (2′-MOE) and locked nucleic acid (LNA)^35^, are used to further increase stability, enhance target binding, and confer less toxicity than unmodified designs. Based on our results that AC104041.1 knockdown could inhibit tumorigenesis which indicated that AC104041.1 may as a potential novel target for HSNCC treatment. Interestingly, we designed LNA-modified ASO-targeting AC104041.1 and observed that it exhibited effectively anti-tumor activity in vitro and in vivo, suggesting that AC104041.1 specific LNA-ASO may as a novel anticancer drug to prevent tumor growth and metastasis in HNSCC.

By using bioinformatics analysis and diverse verification, we demonstrated that Wnt2B as the direct target of AC104041.1/miR-6817-3p axis, and AC104041.1 promoted activation of Wnt/β-catenin signaling through Wnt2B expression. Because there are no specific inhibitors of Wnt2B currently and salinomycin has CSC-specific inhibitory effect through the block for the phosphorylation of lipoprotein receptor-related protein 6 (LRP6), a Wnt coreceptor, and subsequently inhibiting Wnt/β-catenin signaling in breast and prostate cancer^36^. Recently, Wang et al. showed that salinomycin has CSC-specific inhibitory effect on neuroblastoma stem cells by binding nucleolin protein^37^, while the effect and mechanisms of salinomycin in HNSCC remains incompletely understood. Here, our data showed that salinomycin significantly inhibited cell proliferation and migration, and enhanced the anti-tumor activity and suppression of nuclear β-catenin signals with AC104041.1 specific LNA-ASO. These results provide a new therapeutic strategy for HNSCC treatment and future investigations should illuminate the targeting effect and in-depth mechanism of the combination therapy.

### Conclusion

In summary, detailed knowledge of an integrated platform combining genome-wide association analysis and clinical patient management is expected to facilitate the identification of cancer-driving biomarkers. We first identified an oncogenic lncRNA AC104041.1, which as a ceRNA for miR-6817-3p, mediates tumor growth and metastasis by regulating Wnt2B and consequently activating the Wnt/β-catenin pathway. Moreover, our effective exploration of ASO-based gene therapy targeting AC104041.1 and combination of salinomycin provide a promising therapeutic strategy for HNSCC. In the longer term, because upregulation of AC104041.1 has been observed in other tumors including lung adenocarcinoma, stomach adenocarcinoma and hepatocellular carcinoma, it is likely that our understanding of the mechanism and anti-tumor activity of AC104041.1 in HNSCC will give the likelihood of successful therapy in different kinds of cancer.

## Materials and methods

### Cell culture and reagents

All cell lines used in this study were purchased from the American Type Culture Collection (ATCC). CAL27 and 293T cells were cultured in Dulbecco’s modified Eagle’s medium (DMEM) supplemented with 10% FBS. SCC4 cells were cultured in DMEM/F12 medium supplemented with 10% FBS and 400 ng/ml hydrocortisone. All media were supplemented with penicillin/streptomycin to avoid bacterial contamination. All cell lines were cultured in a humidified incubator containing 5% CO_2_ at 37 °C. Cell lines were authenticated by short tandem repeats (STR) profiling.

### The Cancer Genome Atlas analysis

The RNA seq data and complete clinical-pathological information (Table S8) for 500 HNSCC tumors was obtained through the TCGA data portal (https://portal.gdc.cancer.gov/). The complete analysis detailed and clinical pathologic characteristics can be found in the Supporting Information.

### Coding potential analysis

To confirm that the lncRNA genes are non-coding as annotated, we used the algorithm CPAT^38^ (http://lilab.research.bcm.edu/cpat) and CPC^39^ (http://cpc.cbi.pku.edu.cn) with the default parameter. For the lncRNA gene with more than one transcript, we only considered it as non-coding if all its transcripts were non-coding.

### MiRNA target prediction and sponge network construction

MiRNA sequences and family information were obtained from TargetScan (http://www.targetscan.org/) and miRBase release 21. The primary transcripts of lncRNA AC104041.1 (ENSG00000259692) isoforms were derived from Ensembl (http://ensembl.org/index.html). The complete procedure of sponge network construction can be found in the Supporting Information.

### HNSCC patients and clinical specimens

HNSCC tumor tissues and normal-adjacent tissues specimens were collected from Nanjing Medical University Affiliated Hospital of Stomatology, China. The clinical HNSCC specimens were conducted under our institutional guidelines and the supervision of the Institutional Review Board of Nanjing Medical University Affiliated Hospital of Stomatology, and written informed consent was obtained from all participants.

### RNA extraction and qPCR

Real-time quantitative PCR was performed in a reaction mix of SYBR Green (Applied Biological Materials, Inc., Canada) with ABI QuantStudio 3 Real-Time PCR System (Applied Biosystems, USA). The relative expression levels were calculated with the 2^[−∆∆Ct]^ method and expressed as “fold change”. The mRNA and lncRNA levels were normalized against GAPDH in cell and tissue lysates, and the human primer sequences list is reported in Table S9. Human miR-7156-3p 5′-primer, miR-516b-5p 5′-primer, miRNA-6817-3p 5′-primer, U6 housekeeping gene 5′-primer, and the universal 3′ miRNA primer were purchased from Applied Biological Materials, Inc. The miRNA levels were normalized against U6 RNA.

### Lentiviral transduction and generation of stable cell lines

Lentiviral vectors (pLenti-CMV-GFP-Puro, addgene) harboring the cDNA sequence of AC104041.1 or pLVX-shRNA2-Puro harboring two different shRNA sequence of AC104041.1 (sequences list is reported in Table S10) with psPAX2 (addgene) and pMD2.G (addgene) were co-transfected into HEK-293T cells using polyethylene imine (PEI, Sigma). The experimental details can be found in the Supporting Information.

### siRNAs and miRNA transfection

Cells were transfected with siRNA and miRNA as described previously^40^. Transfection of siRNA (GenePharma, China, sequences list is reported in Table S10) or miRNA-6817-3p mimics (GenePharma, China, sequences list is reported in Table S10) or inhibitors (GenePharma, China, sequence: 5′-UGCCAUGGAGUCAGAGAGA-3′) was performed using GP-transfect-Mate (GenePharma, China) at a final concentration of 80 nM. Two days later, the indicated cells were detached, and further biochemical assays were performed.

### Antisense oligonucleotides

Treatment with custom LNA-modified antisense oligonucleotides was performed according to the manufacturer’s protocol (GenePharma, China). The custom sequence of the ASO against AC104041.1 (AC104041.1-ASO) used was 5′-UUUCGAGGAGGAGCCAAGATT-3′, and negative-control ASO (control-ASO) sequence was 5′-ACGUGACACGUUCGGAGAATT-3′. Both oligonucleotides contain phosphorothioate backbone and 2′-Ome modifications. ASO were added to cells and the final concentration was 1 μM without the use of a transfection reagent in vitro. For animal study with ASO injection, mice were injected with 10 mg/kg of AC104041.1-ASO or control-ASO via the tail vain, every 2 days for up to 28 days without any delivery system.

### Analysis of cell viability, cell apoptosis, colony-forming ability, and migration

The sample preparation and experimental details can be found in the Supporting Information.

### Subcellular fractionation

Cytosolic and nuclear fractions of SCC4 and CAL27 cells were prepared and collected according to the instructions of the PARIS Kit (Invitrogen). RNA samples were quantified by qRT-PCR described above. GAPDH and U6 were used as control for cytosolic RNA and nuclear RNA, respectively.

### RNA fluorescence in situ hybridization (FISH)

To detect the subcellular location of AC104041.1 in SCC4 and CAL27 cells, FISH assay was performed as previously described^41^. The complete procedure of sample preparation and experimental details are provided in the Supporting Information.

### Luciferase reporter assay

For the dual-luciferase reporter assay, the fragment of AC104041.1 or Wnt2B containing miR-6817-3p putative target sites were amplified and subcloned into the pmirGLO Dual-Luciferase reporter vector (Promega). The relative luciferase activity was normalized to Renilla luciferase activity 48 h after transfection by the Dual-Luciferase Reporter Assay System (Promega).

### Biotin-miRNA pull-down assay

The miRNA pull-down assay was performed as previously described^42^. The complete procedure of sample preparation and experimental details are provided in the Supporting Information.

### RNA immunoprecipitation (RIP) assays

RIP experiments were performed using the Magna RIP RNA-Binding Protein Immunoprecipitation Kit (Millipore). The antibody against Ago2 (Millipore) and isotype control (IgG) were used according to the manufacture’s protocol. Total RNA (input control) and precipitation with the anti-Ago2 were assayed simultaneously. The co-precipitated RNAs were detected by qRT-PCR.

### Immunoblot assays

The complete procedure of sample preparation and experimental details are provided in the Supporting Information, all the antibodies information were listed in the Table S11.

### Immunofluorescence assays

The complete procedure of sample preparation and experimental details are provided in the Supporting Information.

### Animal studies

For xenograft assays, female 4–6-week-old BALB/c nude mice were randomly divided into different groups as described above. All the animal experiments were complied with IACUC (Institutional Animal Care and Use Committee) regulations and approved by the Ethics Committee of China Pharmaceutical University Permit Number SYXK2012-0035. The complete demand of mouse and experimental details are provided in the Supporting Information.

### Immunohistochemistry analysis

The complete procedure of sample preparation and experimental details are provided in the Supporting Information.

### Statistical analysis

All statistical tests were performed using SPSS20.0 (IBM) and GraphPad Prism 7 software. Student’s unpaired *t*-test (two-sided) was used to analyse the variance between groups. Significant *P* values are indicated with asterisks as follows: *P < 0.05, **P < 0.01, and ***P < 0.001.

## Supplementary information

Supplementary Information

Supplementary Fig S1

Supplementary Fig S2

Supplementary Fig S3

Supplementary Fig S4

Supplementary Fig S5

## Data Availability

The TCGA data referenced in the study are available in a public repository from the National Cancer Institute Cancer Genome Atlas website (https://cancergenome.nih.gov). All other data supporting the findings of this study are available within the article and its Additional files.
