# Supplementary material for: Antisense oligonucleotides targeting lncRNA AC104041.1 induces antitumor activity through Wnt2B/β-catenin pathway in head and neck squamous cell carcinomas
Source: Cell Death Dis. 2020 Aug 13;11(8):672. doi: 10.1038/s41419-020-02820-3 (PMC7443144; doi:10.1038/s41419-020-02820-3)
Supplement: Supplementary file 1 — Supplementary Information [file 41419_2020_2820_MOESM1_ESM.docx]

**Supplementary Information for**

**Antisense oligonucleotides targeting lncRNA AC104041.1 induces**

**antitumor activity through Wnt2B/β-catenin pathway**

**in head and neck squamous cell carcinomas**

**Table of Contents**

**Materials and Methods**

**Table S1** The top 30 differentially expressed lncRNAs in 500 HNSCC patients from TCGA cohort.

**Table S2** The deregulated lncRNAs significantly associated with overall survivial (OS) of patients in the training set and validation set.

**Table S3** The ROC analyses of deregulated lncRNAs signatures in the training set and validation set.

**Table S4** Clinical pathologic characteristics of the independent validation cohort of 94 HNSCC patients.

**Table S5** Univariate and multivariate Cox regression analysis of the lncRNA signature and overall survival in HNSCC patients.

**Table S6** The average expression levels of miRNAs in the interaction network and correlation with lncRNA AC104041.1.

**Table S7** The potential targets of miR-6817-3p were predicted by using TargetScan release 7.2 (Top100).

**Table S8** Clinical pathologic characteristics of 500 HNSCC patients in the original TCGA cohort.

**Table S9** Primers sequence used in the qRT-PCR assays for indicated genes.

**Table S10** The target shRNA and siRNA sequences used in the study.

**Table S11** Primary antibodies used in this study

**Supplementary Figure legends**

**Materials and Methods**

**The Cancer Genome Atlas analysis**

The obtained reads (counts) were normalized to their library sizes and transcript length (RPKM normalization). Using these normalized counts, the significance of differential expression between the normal samples and corresponding solid tumor was obtained using the Wilcoxon signed-rank non-parametric test and corrected for multiple testing with Benjamini- Hochberg’s method (FDR). We used log-fold change ± 1 and FDR < 1E-004 for tumor and matched normal samples to be considered as differentially expressed lncRNAs, these cancer-associated lncRNAs were further subjected to clinical analysis.

**MiRNA target prediction and sponge network construction**

To construct the lnc-AC104041.1 as the ceRNA in the miRNA/mRNA regulatory networks, we presented a comprehensive computational approach as follows: (1) To screen the target miRNAs of AC104041.1, we analyzed the binding energy of all miRNAs (<http://www.mirbase.org/index>. shtml) located on the AC104041.1 transcript. To increase the stringency of prediction, the minimum free energy of the hybridization value was set to -20 kcal/mol as a cut-off. Additionally, binding site conservation was detected using the targetscan _50 software. (2) The secondary structure of RNA targeting sites was deconstructed and analyzed by PITA and RNA fold. The ΔΔG values below -10 kcal/mol were set and the prediction of secondary structure of binding sites was analysed using RNA fold. (3) The target mRNAs of miRNAs were predicted using TargetScan release 7.2. We exported these results as the nodes and edges of a concept association network and visualized the network using Cytoscape version 3.3.0.

**RNA fluorescence in situ hybridization (FISH)**

Briefly, SCC4 and CAL27 cells grown on the slides were washed with PBS and fixed in 4% paraformaldehyde for 5 min at room temperature, and then dehydrated by immersion in an ethanol gradient and air dried, the AC104041.1 probe was diluted in 250 μL pre-warmed in hybridization buffer. Each sample was covered with 50-100 μL diluted probe and incubated in a humidified hybridization chamber at 50 °C overnight, 10% formamide and 2× SSC at 37 °C overnight followed by thorough washing. The images were captured using a confocal microscope (ZEISS, AiryScan LSM 800).

**Biotin-miRNA pulldown assay**

In brief, cells were transfected with biotin-miR-6817-3p or biotin-scramble, biotin-AC104041.1 or biotin-antisense probe (GenePharma). After 24 h, cells were lysed in RIP buffer (150 mM KCl, 25 mM Tris (pH 7.4), 0.5 mM dithiothreitol, 0.5% NP-40, protease inhibitors cocktail and RNase inhibitors). Cell lysates were mixed with streptavidin magnetic beads in RIP buffer and incubated at 4 °C for 4 h. Beads were washed five times with RIP buffer. RNA bound to the beads was isolated using Trizol reagent (Invitrogen) and quantified by qRT-PCR.

**Lentiviral transduction and generation of stable cell lines**

After 48 h, supernatant was collected and filtrated through a 0.45 μm filter to remove the cell debris and concentrated with the Fast-Trap Lentivirus Purification and Concentration Kit (Millipore), following the manufacturer’s protocol. Two days after infection, puromycin (Invitrogen) was added at a final concentration of 2 µg/ml to select stable cell lines.

**Analysis of cell viability, cell apoptosis, colony-forming ability and migration**

For cell viability analyses, 5×10^3^ cells were seeded in 96-well plates and cultured for 3 days. After different selected time, cell viability was measured using MTT assays (Sigma). For cell apoptosis analysis, cells were stained with Annexin V and propidium iodide using the AnnexinV-FITC Apoptosis Detection kit (Multisciences, China), and the percentage of apoptotic cells was determined by flow cytometry (Miltenyi Biotec). For colony formation assays, 500 cells were seeded in 6-well plates and allowed to grow until visible colonies formed in complete growth medium (2 weeks). Cell colonies were fixed with 4% paraformaldehyde, stained with crystal violet and counted. The cell migration assay was performed in transwell chambers (Millipore). In brief, 5×10^3^ cells were resuspended in serum-free medium and cultured in the upper chamber. DMEM medium containing 10% FBS was added to the lower chambers as a chemoattractant. After 48 h, cells that migrated through the filters were fixed with methanol and stained with crystal violet. Representative fields were photographed, and the number of migrated cells per field was counted.

**Immunoblot assays**

Protein extracts from cells or tumor tissues were prepared using RIPA lysis buffer (Beotime, China) supplemented with protease and phosphatase inhibitors. Total protein was subjected to SDS-PAGE anlaysis and transferred to a PVDF membrane (Millipore). Membranes were incubated overnight at 4 °C with primary antibodies and subsequently incubated with the HRP-conjugated secondary antibody, detected with the Western Blot Detection kit (Tanon, China) and analyzed using the Image J programme.

**Immunofluorescence assays**

Cells were seeded onto coated cover slips to grow for 24 h and fixed with 4% paraformaldehyde for 15 min at room temperature, and blocked with 10% goat normal serum + 0.1% Triton X-100 in PBS for 1 h. Cells were incubated with primary antibodies overnight at 4 °C, followed by incubation with Alexa Fluor 488 goat anti-rabbit IgG (H+L) and Alexa Fluor 647 goat anti-mouse IgG (H+L). Images were captured using a confocal microscope (IX53, Olympus, Center Valley, PA).

**Animal studies**

A total of 1×10^7^ cells were injected subcutaneously into both sides of each mice. Tumor size was measured three times a week and tumor volume was calculated according to this formula: tumor volume (mm^3^) = length × width^2^ × 0.5 for 8 weeks after injection.

To examine the roles of AC104041.1 in lung and liver metastasis models, 1×10^6^ cells were injected into BALB/c nude mice via the tail vein. Tumor metastasis was monitored twice a week using the IVIS Spectrum living imaging system (Perkin Elmer). The lungs, livers and cancers for the xenograft model were imaged by IVIS Spectrum and dissected for pathological observation with HE staining.

For PDX model establishment, HNSCC tumor tissues were placed in DMEM/F-12 medium and cut into small pieces (diameter, 0.8-1.5 mm) using dissection scissors. These tumor tissues were subcutaneously injected into the flanks of female NOD-scid-gamma (NSG) mice (5-6 weeks). When tumor size reached 1.5 cm^3^, the tumors were dissected, processed and reinjected for expansion (passage 1, or P1). This process was repeated to expand the tumor tissue. For the co-clinical trial, we used P4 passage to perform further studies. At the end point of the experiment, mice were humanely euthanized as approved by the Ethics Committee of China Pharmaceutical University.

**Immunohistochemistry analysis**

IHC for target molecules (Ki-67) was performed on serial sections from tumor tissues of nude mice xenografts and HNSCC patients. Tissue sections (5 μm thick) were formalin-fixed, paraffin- embedded and incubated with primary antibodies that were diluted in blocking buffer overnight at 4 °C. After washing twice with 0.1% PBS-Tween, slides were incubated with the secondary antibody (Abcam). Finally, sections were incubated with DAB reagent (Dako) and captured in 10 random fields under a ×400 magnification (IX53, Olympus).

**Table S1.** The top 30 differentially expressed lncRNAs in 500 HNSCC patients from TCGA cohort.

| Feature | Name | Log (FC) | Log CPM | LR | P value | FDR |
| --- | --- | --- | --- | --- | --- | --- |
| ENSG00000244128 | LINC01322 | 7.4881 | 0.2873 | 96.9627 | 7.06447E-23 | 4.92707E-21 |
| ENSG00000250874 | CTC-480C2.1 | 7.3029 | -2.1274 | 175.4626 | 4.74451E-40 | 4.89001E-37 |
| **ENSG00000259692** | **AC104041.1** | **7.0745** | **-1.5684** | **165.5882** | **6.80472E-38** | **5.04964E-35** |
| ENSG00000249550 | LINC01234 | 6.8216 | 0.4590 | 109.2704 | 1.41591E-25 | 1.41989E-23 |
| ENSG00000259672 | RP11-69G7.1 | 6.6722 | -0.0732 | 68.5373 | 1.24498E-16 | 3.14243E-15 |
| ENSG00000250920 | RP11-297P16.4 | 6.2632 | 2.2412 | 49.0574 | 2.4858E-12 | 3.03398E-11 |
| ENSG00000249395 | CASC9 | 6.1431 | 1.6829 | 111.2230 | 5.28764E-26 | 5.83906E-24 |
| ENSG00000223812 | RP11-197K6.1 | 6.1183 | -0.8123 | 54.5770 | 1.49475E-13 | 2.288E-12 |
| ENSG00000237445 | CTA-520D8.2 | 5.9787 | -1.7337 | 81.2380 | 2.0011E-19 | 8.15922E-18 |
| ENSG00000230838 | LINC01614 | 5.8498 | -1.0983 | 124.2328 | 7.49193E-29 | 1.49452E-26 |
| ENSG00000228742 | RP5-884M6.1 | 5.8157 | -0.2102 | 119.5223 | 8.04841E-28 | 1.26537E-25 |
| ENSG00000229967 | RP11-366F6.2 | 5.7127 | -2.3170 | 58.4898 | 2.04352E-14 | 3.62096E-13 |
| ENSG00000277268 | RP11-445F12.1 | 5.5816 | -1.7519 | 79.0816 | 5.95978E-19 | 2.27971E-17 |
| ENSG00000267284 | RP11-397A16.1 | 5.4802 | -1.4178 | 166.9061 | 3.50706E-38 | 2.71095E-35 |
| ENSG00000270372 | RP11-109M17.2 | 5.4323 | -2.3407 | 66.1461 | 4.18709E-16 | 9.61372E-15 |
| ENSG00000272763 | RP11-357H14.17 | 5.4154 | 0.1684 | 80.4917 | 2.91932E-19 | 1.17133E-17 |
| ENSG00000264464 | RP11-110H1.8 | 5.3246 | -2.1772 | 53.2775 | 2.896E-13 | 4.20725E-12 |
| ENSG00000250564 | RP11-215P8.4 | 5.2773 | -2.2071 | 65.9505 | 4.62385E-16 | 1.04996E-14 |
| ENSG00000244675 | AC108676.1 | 5.2597 | 1.0074 | 88.8338 | 4.29412E-21 | 2.28265E-19 |
| ENSG00000235385 | GS1-600G8.5 | 5.2566 | 0.3020 | 78.0312 | 1.01423E-18 | 3.7557E-17 |
| ENSG00000251281 | CTD-2066L21.2 | 5.1822 | -2.5075 | 83.2289 | 7.30811E-20 | 3.22043E-18 |
| ENSG00000233532 | LINC00460 | 5.1352 | -1.0217 | 115.8071 | 5.23856E-27 | 6.65656E-25 |
| ENSG00000249001 | RP11-742B18.1 | 5.1351 | -0.3641 | 135.2396 | 2.92641E-31 | 8.75657E-29 |
| ENSG00000256151 | RP11-76C10.5 | 5.1326 | -1.0203 | 82.6914 | 9.59171E-20 | 4.1576E-18 |
| ENSG00000253706 | RP11-758M4.4 | 5.1169 | 0.2848 | 37.7329 | 8.1125E-10 | 6.49441E-09 |
| ENSG00000225548 | AC098973.2 | 5.0931 | 0.0889 | 44.9211 | 2.0514E-11 | 2.1453E-10 |
| ENSG00000229618 | AC011288.2 | 5.0403 | -0.9615 | 92.6843 | 6.13383E-22 | 3.6708E-20 |
| ENSG00000228630 | HOTAIR | 5.0359 | -2.3162 | 57.6133 | 3.19059E-14 | 5.4858E-13 |
| ENSG00000248112 | RP11-78C3.1 | 4.9509 | -2.9370 | 64.2274 | 1.10858E-15 | 2.36394E-14 |
| ENSG00000267123 | CTD-2357A8.3 | 4.8533 | -0.9802 | 95.1681 | 1.74889E-22 | 1.1188E-20 |

**Table S2.** The deregulated lncRNAs significantly associated with OS of patients in the training set and validation set.

| Gene name | Genomic coordinates | Training set | | |  | Validation set | | |
| --- | --- | --- | --- | --- | --- | --- | --- | --- |
|  |  | *P*-value | HR | 95% CI of HR |  | *P*-value | HR | 95% CI of HR |
| ST3GAL4-AS1 | chr11:126340889 -126355587 | 2.12E-03 | 2.258 | 0.409-3.518 |  | 3.57 E-04 | 3.57 | 2.400-4.176 |
| AC103702.2 | chr17: 48635923- 48647023 | 6.204E-03 | 2.737 | 0.023-3.066 |  | 8.58 E-04 | 3.333 | 0.40-3.793 |
| LINC00460 | chr13:106376563-  106378217 | 2.24E-03 | 3.056 | 1.251-3.494 |  | 5.05 E-03 | 2.804 | 1.141-3.130 |
| LINC00668 | chr18:6919496-  6929966 | 4.64E-03 | 2.831 | 1.185-3.271 |  | 0.0118 | 2.519 | 1.212-2.884 |
| LINC00958 | chr11:12961842-  12989548 | 0.01023 | 2.068 | 0.043-2.645 |  | 2.96 E-03 | 1.872 | 0.105-2.017 |
| AC104041.1 | chr11:12961842-  12989548 | 6.75E-03 | 2.709 | 2.176-3.612 |  | 2.47 E-04 | 2.665 | 2.181-3.544 |
| AP005230.1 | chr18:1883524-  2489426 | 0.0112 | 1.618 | 1.109-3.318 |  | **0.067** | 1.061 | 0.084-1.488 |
| MIR31HG | chr9:21455642-  21559669 | 5.96E-03 | 1.865 | 1.017-2.038 |  | **0.209** | 1.152 | 1.044-1.845 |
| AC005537.2 | chr7:42954135-  43113931 | 5.05E-03 | 1.804 | 1.141-3.130 |  | **0.121** | 1.309 | 1.038-1.738 |
| AC126768.2 | chr5:1933863-  1959176 | 9.23E-03 | 2.203 | 2.058-2.649 |  | **0.056** | 1.499 | 1.125-2.133 |
| CTC-276P9.4 | chr5:135034521-  135035894 | 8.43E-03 | 1.634 | 0.597-2.608 |  | 0.011 | 2.241 | 0.211-2.613 |
| RP11-7K24.3 | chr5:135034521-  135035894 | 8.29E-03 | 2.241 | 1.024-2.939 |  | 7.12 E-03 | 1.619 | 1.024-2.437 |

**Table S3.** The ROC analyses of deregulated lncRNAs signatures in the training set and validation set.

| Gene name | Training set | |  | Validation set | |
| --- | --- | --- | --- | --- | --- |
|  | AUC | *P*-value |  | AUC | *P*-value |
| ST3GAL4-AS1 | 0.689 | 0.001 |  | 0.702 | 0.005 |
| AC103702.2 | 0.560 | 0.035 |  | 0.517 | 0.137 |
| LINC00460 | 0.677 | 0.003 |  | 0.685 | 0.000 |
| LINC00668 | 0.539 | 0.028 |  | 0.558 | 0.206 |
| LINC00958 | 0.692 | 0.001 |  | 0.631 | 0.004 |
| **AC104041.1** | **0.713** | 0.000 |  | **0.748** | 0.001 |
| CTC-276P9.4 | 0.562 | 0.027 |  | 0.592 | 0.069 |
| RP11-7K24.3 | 0.609 | 0.006 |  | 0.576 | 0.320 |

**Table S4.** Clinical pathologic characteristics of the independent validation cohort of 94 HNSCC patients.

| Characteristic | Independent cohort (n = 94) |
| --- | --- |
| Age(years) |  |
| Median | 63 |
| Range | 38-84 |
| Gender |  |
| Female | 43 (45.7%) |
| Male | 51 (54.3%) |
| Vital status |  |
| Living | 75 (79.8%) |
| Dead | 19 (20.2%) |
| OS time(days) |  |
| Median | 765 |
| Range | 110-998 |
| Tumor site |  |
| tongue | 31 (33.0%) |
| Oral cavity | 43 (45.7%) |
| Tonsil | 5 (5.3%) |
| Larynx | 15 (16.0%) |
| Clinical stage |  |
| Stage I- II | 70 (74.5%) |
| Stage III- IV | 24 (25.5%) |
| Clinical T |  |
| T1-T2 | 35 (37.2%) |
| T3-T4 | 59 (62.8%) |
| Clinical N |  |
| N0-N2 | 69 (73.4%) |
| N3- N4 | 25 (26.6%) |
| Clinical M |  |
| M0-M1 | 79 (84.0%) |
| M4 | 15 (16.0%) |
| HPV status |  |
| Positive | 18 (19.1%) |
| Negative | 37 (39.4%) |
| Unknown | 39 (41.5%) |

**Table S5.** Univariate and multivariate Cox regression analysis of the lncRNA signature and overall survival in HNSCC patients.

| Covariates | Univariate analysis | |  | Multivariate analysis | |
| --- | --- | --- | --- | --- | --- |
| Training set | HR (95% Cl) | *P*-value |  | HR (95% Cl) | *P*-value |
| Age (>60/≤60) | 1.958 (0.582-3.385) | 0.083 |  | 1.581(0.471-2.862) | 0.183 |
| Gender (Female/male) | 1.894 (0.719-3.284) | 0.091 |  | 1.647(0.502-2.874) | 0.102 |
| Stage (I- II vs III-IV) | 2.192(0.495-3.201) | **0.026** |  | 1.785(0.619-2.581) | 0.069 |
| TNM (I- II vs III) | 2.327(0.837-3.194) | **0.018** |  | 2.018(0.813-2.485) | 0.057 |
| HPV status (P/N) | 1.846(0.285-2.318) | 0.072 |  | 1.573(0.391-2.302) | 0.080 |
| AC104041.1 expression level | 3.824(1.028-4.892) | **0.0014** |  | 3.290(1.203-3.981) | **0.0020** |
| Validation set |  |  |  |  |  |
| Age (>60/≤60) | 1.825 (0.573-3.219) | 0.092 |  | 1.537(0.496-2.529) | 0.216 |
| Gender (Female/male) | 1.738 (0.649-3.154) | 0.082 |  | 1.802(0.524-2.931) | 0.163 |
| Clinical Stage (I- II vs III-IV) | 2.373(0.751-3.368) | **0.017** |  | 1.825(0.631-2.693) | 0.058 |
| TNM (I- II vs III) | 2.416(0.797-3.263) | **0.006** |  | 2.149(0.848-2.852) | 0.062 |
| HPV status (P/N) | 1.822(0.469-2.415) | 0.079 |  | 1.624(0.464-2.520) | 0.087 |
| AC104041.1 expression level | 3.617(2.106-4.635) | **0.0021** |  | 3.175(1.325-4.247) | **0.003** |

Cl, confidence interval; HR, hazard ratio. Statistically significant (**P*<0.05 and ***P*<0.01)

**Table S6.** The average expression levels of miRNAs in the interaction network and correlation with lncRNA AC104041.1.

| Gene | mfe of binding site | Group with low  expressed AC104041.1 | Group with high expressed AC104041.1 | *P* value | Spearman correlation coefficients |
| --- | --- | --- | --- | --- | --- |
| miR-7156-3p | -24.71 | 4.283 | 1.985 | 1.27E-03 | -0.761 |
| miR-516b-5p | -22.4 | 3.841 | 1.911 | 1.65E-02 | -0.677 |
| miR-6817-3p | -20.69 | 5.873 | 1.035 | 5.92E-03 | -**0.885** |

mfe, minimum free energy

**Table S7.** The potential targets of miR-6817-3p were predicted by using TargetScan release 7.2 (Top100).

| Target gene | Transcript ID | Total sites | 8mer sites | 7mer-m8 sites | 7mer-A1 sites | 6mer sites | Total context++ score |
| --- | --- | --- | --- | --- | --- | --- | --- |
| TNFRSF13B | ENST00000261652.2 | 14 | 2 | 6 | 6 | 6 | -3.37 |
| CACNG8 | ENST00000270458.2 | 11 | 0 | 2 | 9 | 5 | -2.52 |
| C22orf46 | ENST00000402966.1 | 9 | 2 | 4 | 3 | 1 | -2 |
| SLC9A3R2 | ENST00000424542.2 | 6 | 3 | 0 | 3 | 1 | -1.68 |
| ZNF233 | ENST00000592581.1 | 10 | 0 | 8 | 2 | 0 | -1.47 |
| WNT2B | ENST00000369686.5 | 4 | 0 | 1 | 3 | 6 | -1.17 |
| TLCD2 | ENST00000330676.6 | 6 | 3 | 1 | 2 | 1 | -1.16 |
| CD300E | ENST00000392619.1 | 13 | 2 | 2 | 9 | 3 | -1.08 |
| ZNF655 | ENST00000425063.1 | 5 | 1 | 2 | 2 | 1 | -1.07 |
| TVP23C | ENST00000225576.3 | 7 | 0 | 0 | 7 | 5 | -1.06 |
| F7 | ENST00000375581.3 | 4 | 2 | 1 | 1 | 0 | -1.14 |
| ZNF256 | ENST00000598928.1 | 4 | 0 | 4 | 0 | 1 | -1.02 |
| EMX2 | ENST00000442245.4 | 6 | 1 | 0 | 5 | 1 | -0.96 |
| WSCD2 | ENST00000332082.4 | 8 | 2 | 1 | 5 | 0 | -0.92 |
| GRIN2B | ENST00000609686.1 | 9 | 1 | 5 | 3 | 6 | -0.91 |
| ZNF23 | ENST00000497160.1 | 3 | 0 | 1 | 2 | 0 | -0.91 |
| BTN2A1 | ENST00000429381.1 | 4 | 1 | 3 | 0 | 1 | -0.84 |
| CPLX2 | ENST00000359546.4 | 6 | 1 | 3 | 2 | 0 | -0.83 |
| RP11-178C3.1 | ENST00000591035.1 | 2 | 1 | 0 | 1 | 0 | -0.81 |
| LSAMP | ENST00000490035.2 | 4 | 2 | 0 | 2 | 1 | -0.81 |
| KCNJ6 | ENST00000609713.1 | 3 | 1 | 1 | 1 | 3 | -0.79 |
| ZNF286B | ENST00000285274.5 | 4 | 0 | 2 | 2 | 0 | -0.78 |
| UNC13A | ENST00000519716.2 | 7 | 0 | 2 | 5 | 1 | -0.77 |
| REP15 | ENST00000310791.2 | 2 | 1 | 0 | 1 | 0 | -0.76 |
| MT1F | ENST00000334350.6 | 1 | 1 | 0 | 0 | 0 | -0.75 |
| CTRC | ENST00000375943.2 | 4 | 2 | 0 | 2 | 2 | -0.73 |
| XKR4 | ENST00000327381.6 | 6 | 0 | 2 | 4 | 5 | -0.72 |
| PPP6R1 | ENST00000412770.2 | 5 | 0 | 1 | 4 | 0 | -0.78 |
| PAGR1 | ENST00000609618.1 | 4 | 0 | 0 | 4 | 0 | -0.71 |
| IRX5 | ENST00000394636.4 | 3 | 1 | 0 | 2 | 0 | -0.7 |
| EDN3 | ENST00000311585.7 | 3 | 2 | 0 | 1 | 1 | -0.7 |
| CDHR1 | ENST00000372117.3 | 3 | 2 | 1 | 0 | 2 | -0.97 |
| AL589765.1 | ENST00000442233.2 | 2 | 2 | 0 | 0 | 1 | -0.7 |
| C8orf74 | ENST00000304519.5 | 1 | 1 | 0 | 0 | 0 | -0.69 |
| DLX6 | ENST00000518156.2 | 3 | 1 | 0 | 2 | 0 | -0.69 |
| TIMM10B | ENST00000254616.6 | 2 | 1 | 0 | 1 | 1 | -0.7 |
| GRIK1-AS2 | ENST00000333765.4 | 1 | 1 | 0 | 0 | 0 | -0.68 |
| ZFP69B | ENST00000484445.1 | 3 | 0 | 1 | 2 | 1 | -0.67 |
| ZNF286A | ENST00000395894.2 | 4 | 0 | 2 | 2 | 0 | -0.66 |
| HELLS | ENST00000394036.1 | 3 | 1 | 1 | 1 | 0 | -0.64 |
| DNAH10OS | ENST00000514254.2 | 4 | 2 | 1 | 1 | 2 | -0.63 |
| IKZF1 | ENST00000331340.3 | 4 | 1 | 2 | 1 | 0 | -0.62 |
| Z98049.1 | ENST00000598601.1 | 4 | 1 | 1 | 2 | 0 | -0.62 |
| GPR179 | ENST00000342292.4 | 2 | 1 | 0 | 1 | 0 | -0.62 |
| SMTNL2 | ENST00000338859.4 | 5 | 2 | 0 | 3 | 0 | -0.68 |
| LACC1 | ENST00000325686.6 | 4 | 0 | 0 | 4 | 2 | -0.62 |
| DDX52 | ENST00000349699.2 | 1 | 1 | 0 | 0 | 1 | -0.69 |
| KRT28 | ENST00000306658.7 | 1 | 1 | 0 | 0 | 0 | -0.61 |
| ATP6AP1L | ENST00000380167.4 | 3 | 0 | 0 | 3 | 0 | -0.61 |
| MAP1B | ENST00000296755.7 | 7 | 1 | 1 | 5 | 2 | -0.61 |
| IPO4 | ENST00000354464.6 | 1 | 1 | 0 | 0 | 0 | -0.6 |
| LINC00923 | ENST00000503874.3 | 3 | 0 | 3 | 0 | 0 | -0.6 |
| MGC10955 | ENST00000401851.1 | 1 | 1 | 0 | 0 | 0 | -0.6 |
| ZNF586 | ENST00000396150.4 | 3 | 0 | 3 | 0 | 0 | -0.59 |
| SFTA3 | ENST00000518529.2 | 2 | 1 | 0 | 1 | 0 | -0.59 |
| CLEC12A | ENST00000355690.4 | 2 | 1 | 1 | 0 | 0 | -0.59 |
| ZNF300 | ENST00000427179.1 | 2 | 0 | 1 | 1 | 1 | -0.59 |
| ZNF772 | ENST00000425074.3 | 2 | 1 | 1 | 0 | 0 | -0.58 |
| FAM104B | ENST00000472571.2 | 1 | 1 | 0 | 0 | 0 | -0.58 |
| GDF11 | ENST00000257868.5 | 5 | 1 | 0 | 4 | 1 | -0.62 |
| ZBTB37 | ENST00000367701.5 | 4 | 0 | 0 | 4 | 7 | -0.64 |
| SHOX | ENST00000381578.1 | 5 | 1 | 1 | 3 | 1 | -0.58 |
| THRA | ENST00000450525.2 | 6 | 2 | 0 | 4 | 2 | -0.57 |
| FGG | ENST00000404648.3 | 2 | 1 | 1 | 0 | 0 | -0.59 |
| MYD88 | ENST00000495303.1 | 3 | 1 | 2 | 0 | 1 | -0.58 |
| ELAVL3 | ENST00000359227.3 | 6 | 1 | 1 | 4 | 0 | -0.57 |
| MUC19 | ENST00000454784.4 | 5 | 0 | 5 | 0 | 0 | -0.57 |
| CLLU1 | ENST00000378485.1 | 3 | 1 | 1 | 1 | 0 | -0.56 |
| CCL16 | ENST00000293275.3 | 3 | 0 | 1 | 2 | 1 | -0.56 |
| CUX1 | ENST00000437600.4 | 2 | 0 | 1 | 1 | 1 | -0.56 |
| C3orf79 | ENST00000446603.2 | 1 | 1 | 0 | 0 | 0 | -0.56 |
| FCAMR | ENST00000400962.3 | 2 | 2 | 0 | 0 | 0 | -0.56 |
| TMIGD1 | ENST00000538566.2 | 1 | 1 | 0 | 0 | 0 | -0.56 |
| DDT | ENST00000398344.4 | 1 | 1 | 0 | 0 | 1 | -0.55 |
| KRBOX4 | ENST00000360017.5 | 2 | 1 | 0 | 1 | 0 | -0.55 |
| AL356356.1 | ENST00000538795.1 | 2 | 2 | 0 | 0 | 1 | -0.55 |
| CDH19 | ENST00000540086.1 | 1 | 1 | 0 | 0 | 0 | -0.55 |
| TNIP3 | ENST00000507879.1 | 1 | 1 | 0 | 0 | 0 | -0.55 |
| ECHDC1 | ENST00000528402.1 | 2 | 0 | 2 | 0 | 0 | -0.54 |
| MC2R | ENST00000327606.3 | 5 | 1 | 0 | 4 | 1 | -0.54 |
| ENDOU | ENST00000229003.3 | 2 | 1 | 0 | 1 | 0 | -0.54 |
| GLS2 | ENST00000311966.4 | 2 | 1 | 0 | 1 | 0 | -0.54 |
| SLCO5A1 | ENST00000260126.4 | 2 | 0 | 1 | 1 | 4 | -0.53 |
| LYSMD3 | ENST00000509384.1 | 3 | 1 | 0 | 2 | 0 | -0.53 |
| CCDC18 | ENST00000334652.5 | 3 | 2 | 0 | 1 | 0 | -0.77 |
| LUZP4 | ENST00000371920.3 | 1 | 0 | 1 | 0 | 0 | -0.53 |
| ZNF225 | ENST00000592780.1 | 4 | 0 | 2 | 2 | 0 | -0.52 |
| ZNF514 | ENST00000295208.2 | 3 | 2 | 0 | 1 | 1 | -0.62 |
| HIST1H2AC | ENST00000602637.1 | 1 | 1 | 0 | 0 | 0 | -0.52 |
| MTCP1 | ENST00000369476.3 | 2 | 1 | 0 | 1 | 1 | -0.52 |
| DUSP6 | ENST00000279488.7 | 3 | 1 | 1 | 1 | 0 | -0.6 |
| YIPF7 | ENST00000415895.4 | 1 | 1 | 0 | 0 | 1 | -0.52 |
| RAB7L1 | ENST00000367139.3 | 2 | 1 | 0 | 1 | 0 | -0.51 |
| DAP | ENST00000230895.6 | 6 | 2 | 3 | 1 | 0 | -0.99 |
| SNX7 | ENST00000306121.3 | 1 | 1 | 0 | 0 | 0 | -0.51 |
| RNFT1 | ENST00000442346.2 | 1 | 1 | 0 | 0 | 0 | -0.51 |
| FBLN5 | ENST00000267620.10 | 1 | 1 | 0 | 0 | 2 | -0.57 |
| C1orf105 | ENST00000367725.4 | 1 | 1 | 0 | 0 | 0 | -0.51 |
| ZNF487 | ENST00000437590.2 | 2 | 1 | 0 | 1 | 0 | -0.51 |
| MZT1 | ENST00000377818.3 | 1 | 1 | 0 | 0 | 0 | -0.5 |

**Table S8.** Clinical pathologic characteristics of 500 HNSCC patients in the original TCGA cohort.

| Characteristic | TCGA cohort (n = 500) |
| --- | --- |
| Age (years) |  |
| Median | 61 |
| Range | 20-90 |
| Gender |  |
| Female | 375 (75%) |
| Male | 125 (25%) |
| Vital status |  |
| Living | 350 (70%) |
| Dead | 150 (30%) |
| OS time(days) |  |
| Median | 660 |
| Range | 0-6417 |
| Tumor site |  |
| tongue | 149 (29.8%) |
| Oral cavity | 178 (35.6%) |
| Tonsil | 41 (8.2%) |
| Larynx | 132 (26.4%) |
| Clinical stage |  |
| Stage I- II | 106 (21.2%) |
| Stage III- IV | 394 (78.8%) |
| Clinical T |  |
| T1-T2 | 173 (34.6%) |
| T3-T4 | 327 (65.4%) |
| Clinical N |  |
| N0-N2 | 473 (94.6%) |
| N3- N4 | 27 (5.4%) |
| Clinical M |  |
| M0-M1 | 479 (95.8%) |
| M4 | 21 (4.2%) |
| HPV status |  |
| Positive | 41 (8.2%) |
| Negative | 81 (16.2%) |
| Unknown | 378 (75.6%) |

**Table S9.** Primers sequence used in the qRT-PCR assays for indicated genes.

| Gene name | Forward sequence (5’ to 3’) | Reverse sequence (5’ to 3’) |
| --- | --- | --- |
| AC104041.1 | CCGCCTTGCAGTTTGATCTC | ACTCCCACCCGAATATTGCG |
| Wnt2B | GGCTGCTACCGCTTCTATTT | GGGAGCTTGCTCAGTTTCTTA |
| GAPDH | TGCACCACCAACTGCTTAGC | GGCATGGACTGTGGTCATGAG |

**Table S10.** The target shRNA and siRNA sequences used in the study.

| sh1-AC104041.1 | Sense (5’- 3’) | CGGGATCCCGAGACCGGAGCTGTTCCTATTCGGCCATCTTTCAAGAGACTCTTGAAAGATGGCCGAATAGGAACAGCTCCGGTCTTTTTTTACGGAATTCC |
| --- | --- | --- |
|  | Anti-sense (5’ - 3’) | GGAATTCCGTAAAAAAAGACCGGAGCTGTTCCTATTCGGCCATCTTTCAAGAGTCTCTTGAAAGATGGCCGAATAGGAACAGCTCCGGTCTCGGGATCCCG |
| sh2-AC104041.1 | Sense (5’- 3’) | CGGGATCCCGGTGGAGTCTTCTCACAGAAACAAGGAGAATTCAAGAGATTCTCCTTGTTTCTGTGAGAAGACTCCACTTTTTTTACGGAATTCC |
|  | Anti-sense (5’- 3’) | GGAATTCCGTAAAAAAAGTGGAGTCTTCTCACAGAAACAAGGAGAATCTCTTGAATTCTCCTTGTTTCTGTGAGAAGACTCCACCGGGATCCCG |
| miR-6817-3p mimic | Sense (5’- 3’) | UCUCUCUGACUCCAUGGCA |
|  | Anti-sense (5’- 3’) | CCAUGGAGUCAGAGAGAUU |
| si-Wnt2B | Sense (5’- 3’) | GCUGCUACCGCUUCUAUUUTT |
|  | Anti-sense (5’- 3’) | AAAUAGAAGCGGUAGCAGCTT |

**Table S11.** Primary antibodies used in this study

| Antibodies | Manufacturer | Catalog |
| --- | --- | --- |
| Wnt2B | Abcam | ab178418 |
| β-catenin | Cell Signaling Technology | 8480 |
| phospho-β-catenin (Ser33/37/Thr41) | Cell Signaling Technology | 9561 |
| c-Myc | Cell Signaling Technology | 5605T |
| Vimentin | Cell Signaling Technology | 5741T |
| GAPDH | Multisciences | ab011 |
| Histone H3 | Abcam | ab1791 |
| Ago2 | Abcam | ab32381 |

**Figure. S1 Characterization of differentially expressed lncRNAs in HNSCC using TCGA data sets.**

**A.** Bioinformatic method for the differential lncRNAs in HNSCC patients from TCGA dataset. **B.** ROC curve of AC104041.1 in HNSCC samples from TCGA database (n = 500). The coding potential analysis of AC104041.1 using CPAT (**C**) and PhyloCSF (**D**) databases.

**Figure. S2 Lnc-AC104041.1 promotes the proliferation and metastatic ability of HNSCC cells. A.** qRT-PCR analysis of AC104041.1 in SCC4 cells transfected with two different sh-AC104041.1 vector. Data are mean values ± SD, the experiment was performed in triplicates and repeated three times, ****P* < 0.001 (Student’s *t*-test). **B.** qRT-PCR analysis of AC104041.1 in CAL27 cells transfected with AC104041.1-overexpressing vector. Data are presented as mean values ± SD, ***P* < 0.01 (Student’s *t*-test). Soft agar colony-forming assay of SCC4 cells with AC104041.1 knockdown (**C**) or CAL27 cells with AC104041.1 overexpression (**D**), Data are presented as the mean values ± SD, the experiment was performed in triplicates and repeated three times, ***P* < 0.01 (Student’s *t*-test). **E.** Representative IHC images of Ki-67 from xenograft tumor with AC104041.1 knockdown or overexpression in nude mice. Scale bar represents 20 µm. **F.** Representative images of lung metastasis of indicated mice treated with different cancer cells. Scale bar represents 20 µm. **G.** Bioluminescence signal of SCC4 cells with AC104041.1 knockdown detected using *in vivo* imaging system for 8 weeks (n = 5 mice for each group). Data are presented as the mean values ± SD, ****P* < 0.001 (Student’s *t*-test). **H.** Bioluminescence signal of ectopically expressing AC104041.1 CAL27 cells detected using *in vivo* imaging system for 8 weeks (n = 5 mice for each group). Data are presented as the mean values ± SD, ****P* < 0.001 (Student’s *t*-test).

**Figure. S3 Molecular pathway analysis for mRNA associated with lncRNAs and identification of complementarity between AC104041.1 and miR-6817-3p.**

**A.** Computational strategy of predicting AC104041.1 mediated sponge regulation of protein-coding driver genes in HNSCC. **B.** Gene ontology enrichment analysis for target genes in the AC104041.1 network. **C.** The expression of top three miRNAs in paired normal and tumor tissues from HNSCC patients (n = 25). Each dot represents the average of qPCR results performed in triplicates. Horizontal lines represent mean values. ****P* < 0.001 (paired t-test). **D.** Schematic illustration of pmirGLO-based luciferase reporter constructs containing wild-type AC104041.1 (pmirGLO- AC104041.1-WT) and a mutant reporter construct in which three putative miR-6817-3p binding sites were mutated (pmirGLO-AC104041.1-MU). Mutated bases are depicted in red. **E.** qRT-PCR analysis of miR-6817-3p expression in SCC4 cells transfected with miR-6817-3p mimics (left panel) or CAL27 cells transfected with miR-6817-3p inhibitors. Data are mean values ± SD, the experiment was performed in triplicates and repeated three times, ****P* < 0.001 (Student’s *t*-test). **F.** Luciferase activities were measured in SCC4 cells co-transfected with luciferase reporters containing nothing, AC104041.1 or mutant transcript and miR-6817-3p inhibitors. Data are presented as the mean values ± SEM, the experiment was performed in triplicates and repeated three times, **P* < 0.05, compared with control cells (one-way ANOVA).

**Figure. S4 Wnt2B is a direct downstream target of miR-6817-3p.**

**A, B, C.** Luciferase activity in SCC4 cells co-transfected with luciferase reporter containing different wide-type seed region of top ten predicted targets and miR-6817-3p mimic. Data are presented as the mean values ± SEM, the experiment was performed in triplicates and repeated three times, **P* < 0.05, ***P* < 0.01, compared with control cells (one-way ANOVA). **D.** Schematic illustration of pmirGLO- based luciferase reporter constructs containing wild-type Wnt2B (pmirGLO-Wnt2B-WT) and a mutant reporter construct in which three putative miR-6817-3p binding sites were mutated (pmirGLO-Wnt2B-mut). Mutated bases are depicted in red. **E.** Immunoblotting for Wnt2B enrichment of Ago2 RIP assay relative to IgG or knockdown of Ago2. **F.** Ago2 was pulled down with biotin-labelled AC104041.1 in whole-cell lysates of SCC4 cells. **G.** AGO2 competing assay using AGO2 antibody-mediated RNA-IP in the sh-AC104041.1 and sh-NC groups. RNA levels of precipitated AC104041.1 and Wnt2B are tested using qPCR assay. Data are presented as the mean values ± SEM, ***P* < 0.001 (one-way ANOVA). **H.** Kaplan-Meier analysis of the correlation between Wnt2B expression levels and overall survival of HNSCC in TCGA data (n = 500). The higher expression of Wnt2B is significantly correlated with poor OS (****P* < 0.001, log rank test). **I.** Association of AC104041.1 and Wnt2B with overall survival in HNSCC (n = 500, ****P* < 0.001, log rank test). **J.** Positive correlation between AC104041.1 and Wnt2B levels in HNSCC tissues (n = 500, Spearman correlation r = 0.7867, ****P* < 0.001).

**Figure. S5 The specificity of AC104041.1 ASO in HNSCC cells.**

**A.** qRT-PCR analysis of AC104041.1 expression in SCC4 cells transfected with AC104041.1 ASO. Data are mean values ± SD, the experiment was performed in triplicates and repeated three times, ****P* < 0.001 (Student’s *t*-test). **B.** qRT-PCR analysis of AC104041.1 in SCC4 cells and normal cell (HIOEC: Human normal oral epithelial cell line, 293T: Human Renal Epithelial Cell line). Data are mean values ± SD, the experiment was performed in triplicates and repeated three times, ****P* < 0.001 (Student’s *t*-test). **C.** Cell proliferation assay of HIOEC and 293T cells transfected with AC104041.1 specific LNA-ASO or control ASO (n=5). Data are presented as the mean values±SEM, the experiment was repeated three times, compared with control cells (two-way ANOVA).
