# Supplementary figures and images for: Antisense oligonucleotides targeting lncRNA AC104041.1 induces antitumor activity through Wnt2B/β-catenin pathway in head and neck squamous cell carcinomas
Source: Cell Death Dis. 2020 Aug 13;11(8):672. doi: 10.1038/s41419-020-02820-3 (PMC7443144; doi:10.1038/s41419-020-02820-3)

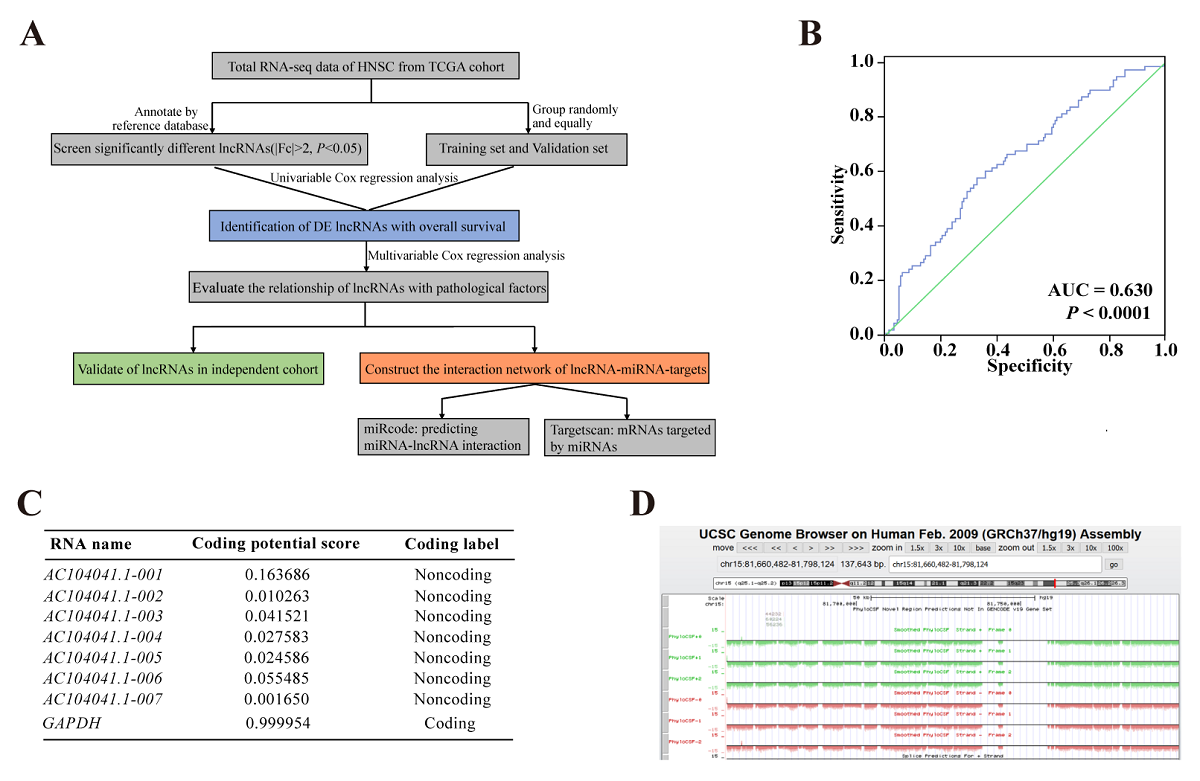

Supplement: Supplementary file 2 — Supplementary Fig S1 [file 41419_2020_2820_MOESM2_ESM.tif]

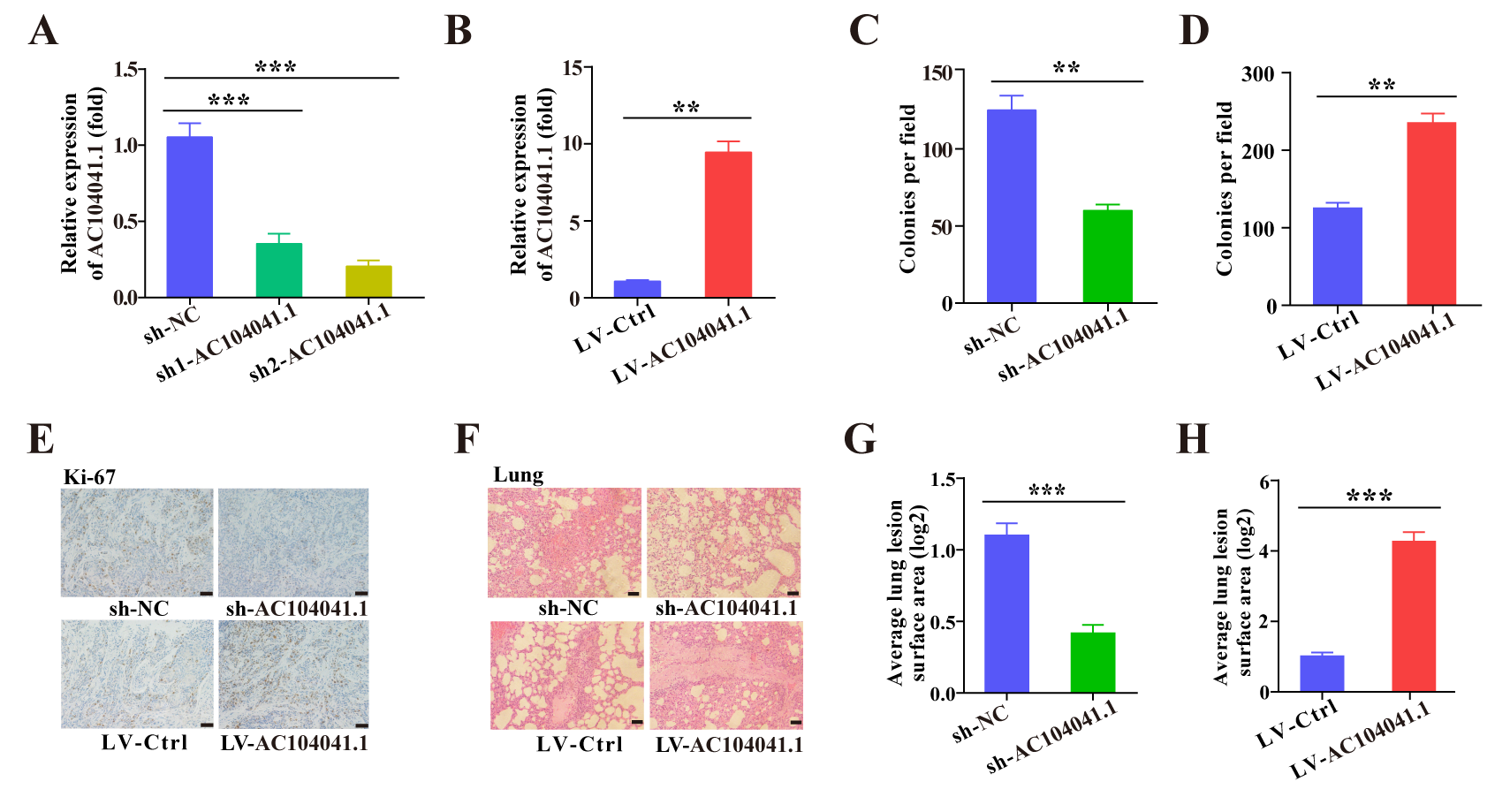

Supplement: Supplementary file 3 — Supplementary Fig S2 [file 41419_2020_2820_MOESM3_ESM.tif]

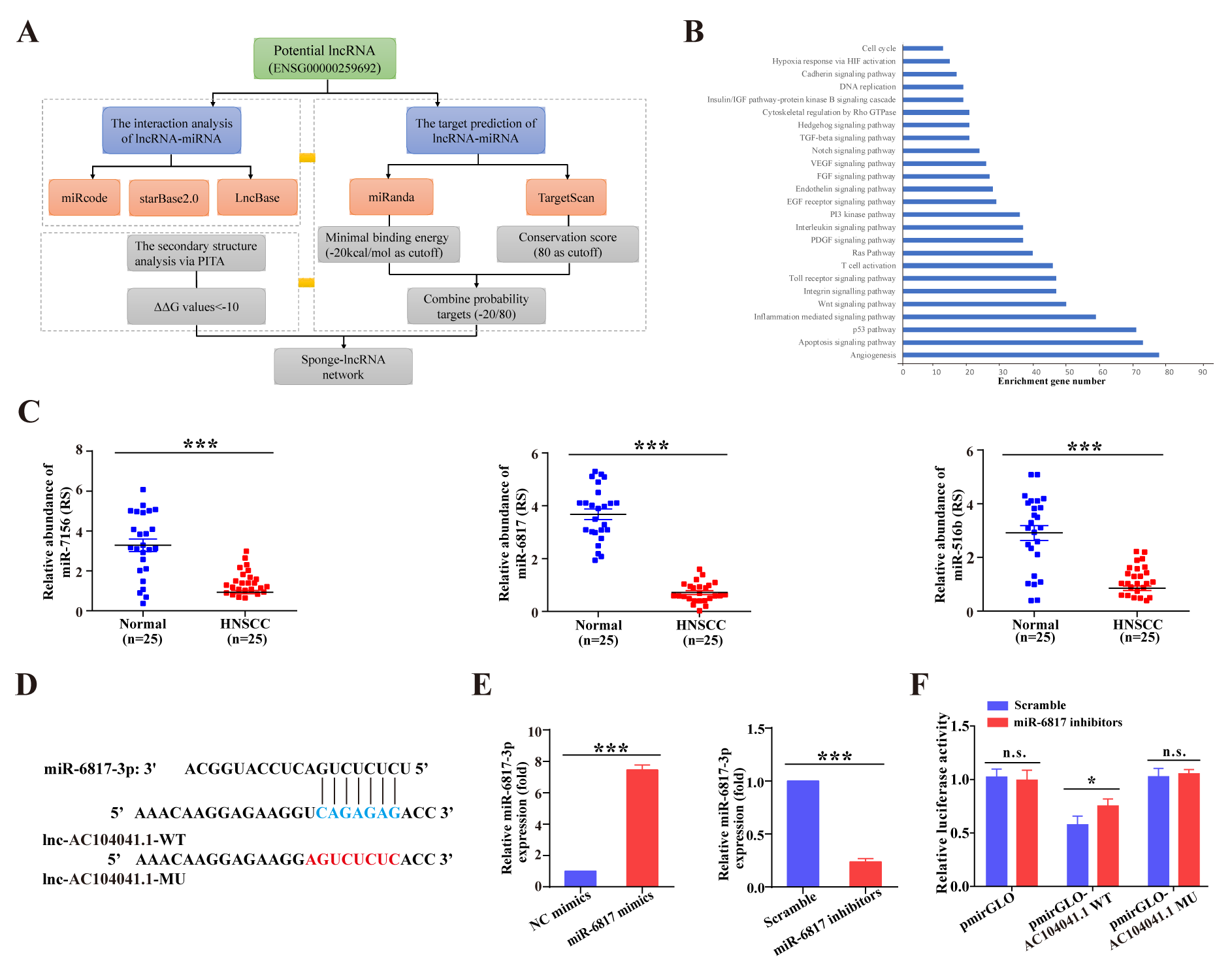

Supplement: Supplementary file 4 — Supplementary Fig S3 [file 41419_2020_2820_MOESM4_ESM.tif]

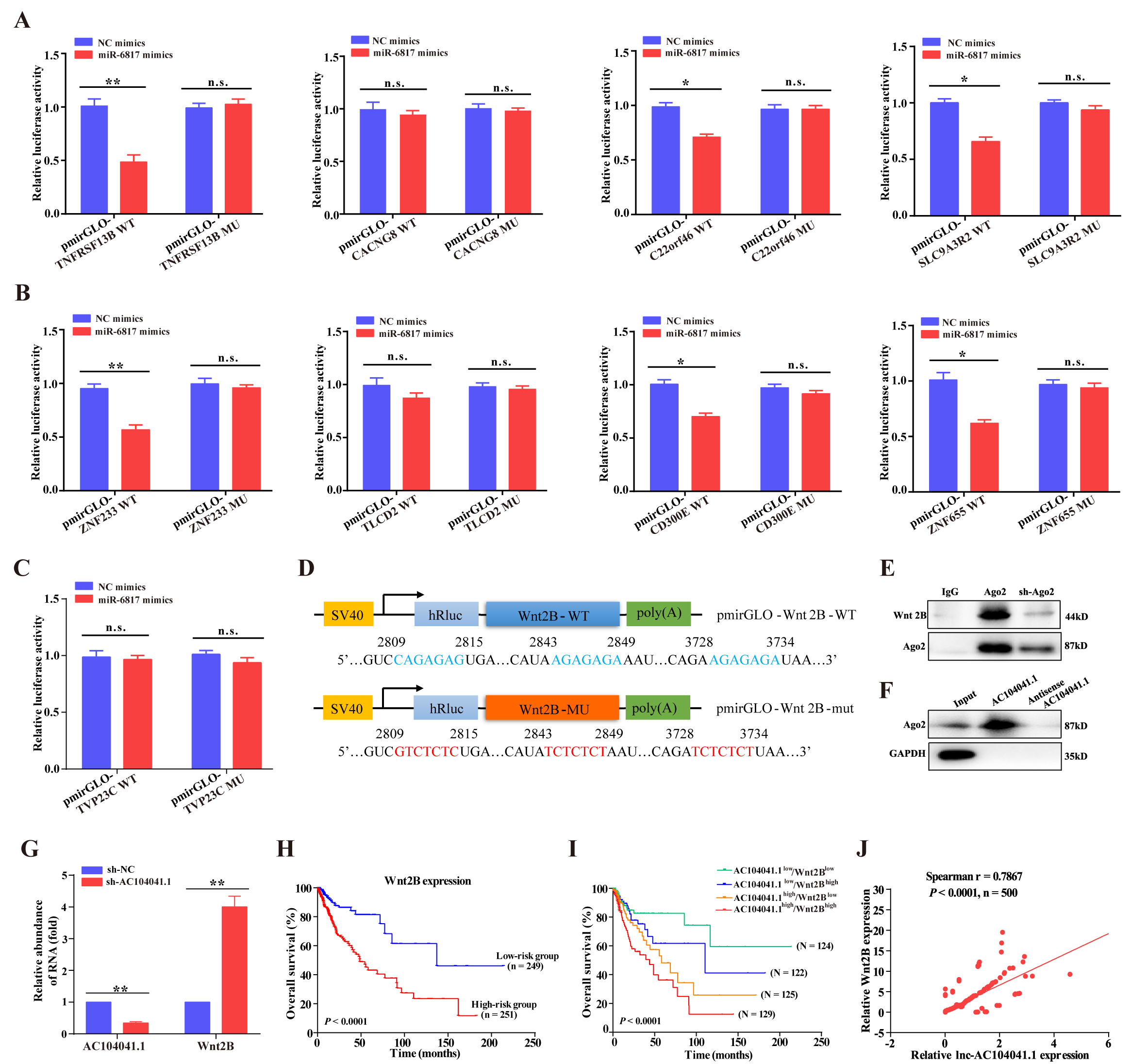

Supplement: Supplementary file 5 — Supplementary Fig S4 [file 41419_2020_2820_MOESM5_ESM.tif]

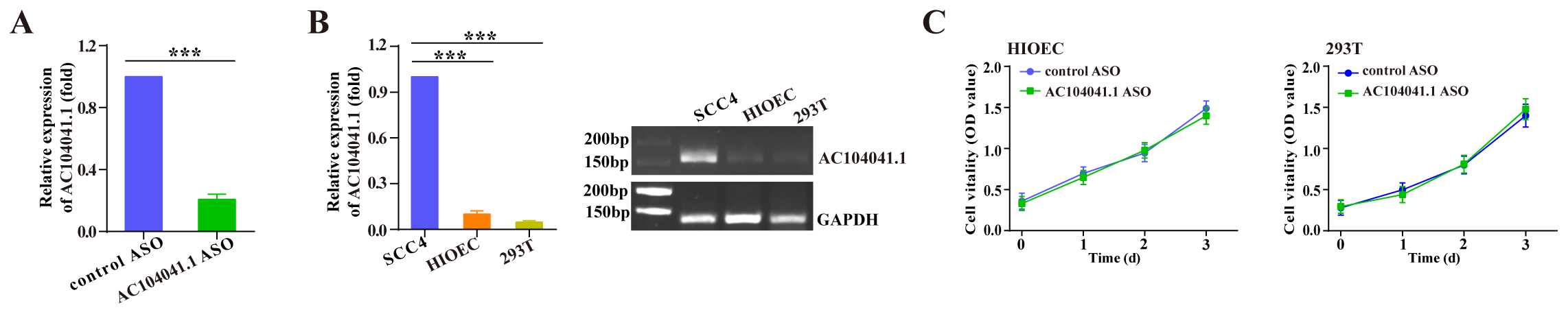

Supplement: Supplementary file 6 — Supplementary Fig S5 [file 41419_2020_2820_MOESM6_ESM.tif]
